# NOD2 signaling in CD11c + cells is critical for humoral immune responses during oral vaccination and maintaining the gut microbiome

**DOI:** 10.1038/s41598-022-12469-x

**Published:** 2022-05-19

**Authors:** B. E. Fox, A. Vilander, Z. Abdo, G. A. Dean

**Affiliations:** Department of Microbiology, Immunology and Pathology, College of Veterinary Medicine and Biomedical Sciences, Colorado State University, Fort Collins, CO 80523 USA

**Keywords:** Immunology, Microbiology, Diseases, Health care

## Abstract

Nucleotide-binding oligomerization domain containing 2 (NOD2) is a critical regulator of immune responses within the gastrointestinal tract. This innate immune receptor is expressed by several cell types, including both hematopoietic and nonhematopoietic cells within the gastrointestinal tract. Vaccination targeting the gastrointestinal mucosal immune system is especially difficult due to both physical and mechanistic barriers to reaching inductive sites. The use of lactic acid bacteria is appealing due to their ability to persist within harsh conditions, expression of selected adjuvants, and manufacturing advantages. Recombinant *Lactobacillus acidophilus* (rLA) has shown great promise in activating the mucosal immune response with minimal impacts on the resident microbiome. To better classify the kinetics of mucosal vaccination with rLA, we utilized mice harboring knockouts of NOD2 expression specifically within CD11c + cells. The results presented here show that NOD2 signaling in CD11c + cells is necessary for mounting a humoral immune response against exogenous antigens expressed by rLA. Additionally, disruption of NOD2 signaling in these cells results in an altered bacterial microbiome profile in both control mice and mice receiving *L. acidophilus* strain NCK1895 and vaccine strain LaOVA.

## Introduction

Addressing the ongoing global threat of mucosally transmitted pathogens will require novel mucosal vaccine platforms to complement or replace existing parenteral vaccines. Rational development of mucosal vaccines requires a comprehensive understanding of how, where and through what cell types a candidate vaccine engages the host immune system. Lactic acid bacteria (LAB), such as *Lactobacillus acidophilus,* provide an attractive platform to deliver selected antigens to mucosal immune inductive sites^1,2^. *L. acidophilus* persists but does not colonize the digestive tract, is generally regarded as safe by the FDA, and offers practical and logistical benefits as a vaccine platform, including inexpensive manufacturing, room temperature storage, and oral delivery^3–5^. *L. acidophilus* expresses microbial-associated molecular patterns that are recognized by the innate immune system and serve as endogenous adjuvants. Importantly, *L. acidophilus* can also be readily engineered to express high levels of exogenous antigens and adjuvants^6–8^. We have previously reported the immunogenicity of recombinant *Lactobacillus acidophilus* (rLA) expressing several antigens^9,10^. However, a mechanistic understanding of mucosal immune activation by rLA in vivo is needed to further optimize this potentially powerful vaccine platform.

Nucleotide-binding oligomerization domain containing 2 (NOD2) is a cytoplasmic pattern recognition receptor in the NOD-like receptor (NLR) family that recognizes the peptidoglycan breakdown product muramyl dipeptide (MDP) from many bacterial species, including *L. acidophilus*^11^. Ligation of NOD2 results in a proinflammatory response mediated by NF-κB, caspase-1, and mitogen-activated protein kinase-signaling (MAPK) cascades^12^. NOD2 is expressed by a variety of cells in the intestinal tract, including hematopoietic (dendritic cells, macrophages, and T and B cells) and nonhematopoietic cells (Paneth cells, goblet cells, and enterocytes)^13^. It has previously been demonstrated that NOD2 signaling in antigen presenting cells (APCs), specifically dendritic cells (DCs), is essential for initiating T_H_2-type and innate T_H_17 immune responses, while NOD2 signaling in Paneth cells leads to the increased production of antimicrobial peptides and a T_H_1-driven response^11,14–16^. Additionally, NOD2 studies have shown that NOD2 signaling is required for the recognition and clearance of pathogens and toxins, including *Streptococcus pneumoniae*, *Citrobacter rodentium*, *Salmonella typhimurium*, *Bacillus anthracis*, and cholera toxin (CT), among others^14,17–20^.

Previously, we investigated the roles of TLR2, NOD2, and caspase-1 in macrophage phagocytosis and activation in response to coculture with rLA in vitro and found that NOD2 is required for a proinflammatory response against rLA. In vivo studies indicated that NOD2 is also critical for antigen-specific mucosal IgA and systemic IgG responses against rLA, but the specific NOD2-expressing cell type(s) responsible were not determined^21^. Here, we utilized Cre-Lox recombination to selectively disrupt NOD2 signaling in CD11c-expressing cells to investigate the role of NOD2 in DCs upon vaccination with rLaOVA, a construct expressing the major histocompatibility complex (MHC) class II epitope from chicken egg ovalbumin (peptide 323–339)^22^. We show that NOD2 signaling in CD11c + DCs is essential for rLA immunogenicity in both mucosal tissues and systemically. In addition, we determined the influence of CD11c-NOD2 signaling by *L. acidophilus* on the composition of the intestinal microbiome and identified several associated key taxa. These results will inform the rational design of rLA vaccine constructs and provide insight into the mechanism and application of *L. acidophilus* as a probiotic.

## Results

### NOD2 signaling in CD11c + cells is required for an OVA-specific humoral response

**Figure 1:**
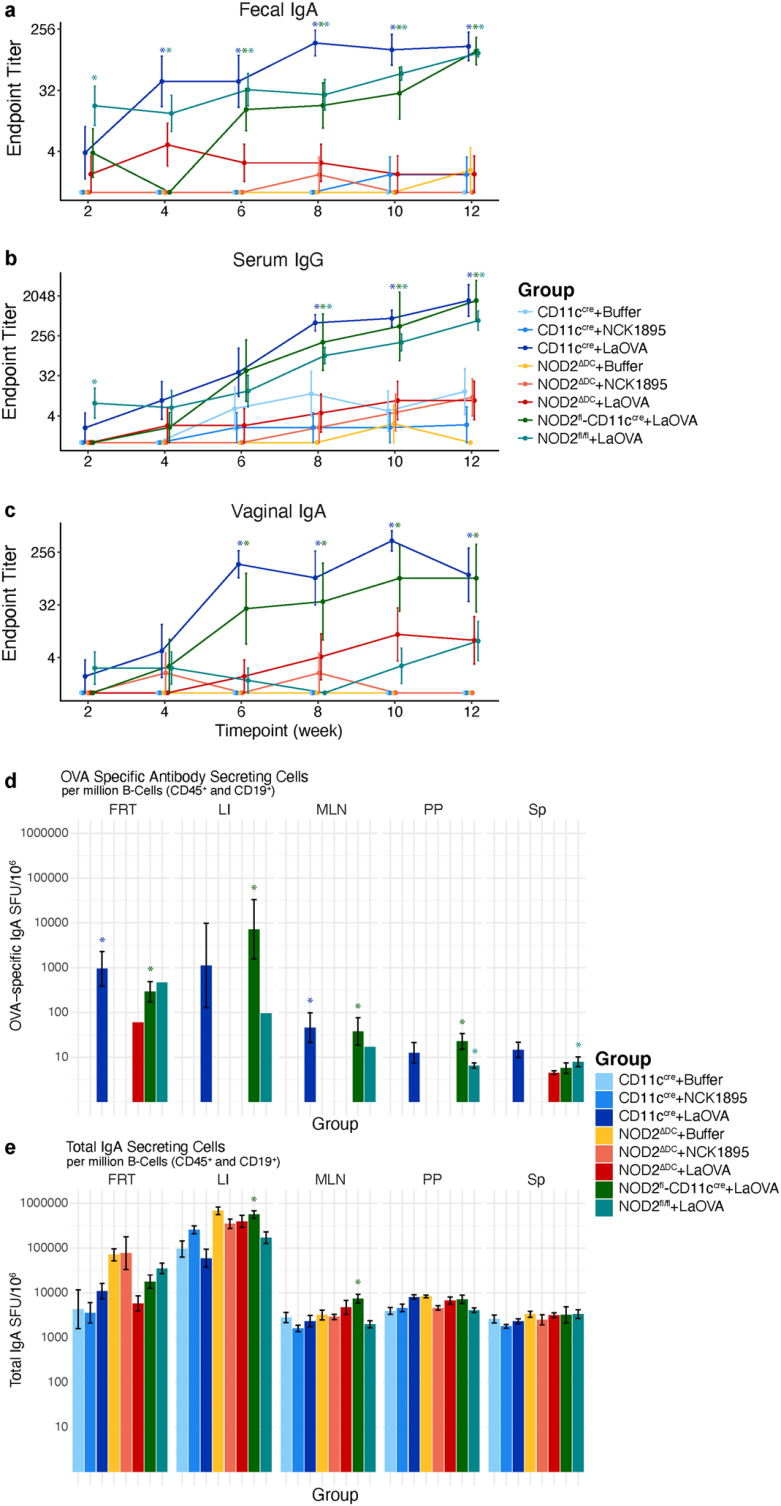
OVA-specific IgA and IgG are dependent on NOD2 signaling in CD11c + cells. CD11c^cre^ and NOD2^ΔDC^ mice were administered buffer only, NCK1895, or LaOVA, and genotype controls were administered LaOVA. Fecal, serum, and vaginal washes were collected every two weeks for detection of OVA-specific antibody production. Fecal IgA (**a**), serum IgG (**b**), and vaginal IgA (**c**) were all measured via ELISA with the OVA_323-339_ peptide. Data are reported as endpoint titers, where the cutoff was determined as described in the methods and represented by the mean and standard error. P values for all pairwise comparisons are shown in Table S1 and adjusted for multiple testing via Benjamini–Hochberg adjustment. At sacrifice, lymphocytes from the female reproductive tract (FRT), large intestine (LI), mesenteric lymph nodes (MLN), Peyer’s patches (PP), and spleen (Sp) were isolated. Cells from each tissue were subjected to OVA-specific (**d**) and total IgA (**e**) ELISpots. Data are represented as spot forming units (SFU) per million antibody secreting cells (defined as CD45^+^CD19^+^7-AAD^-^) for each group. Data are plotted as the mean with standard error, and P values from pairwise comparisons using Benjamini–Hochberg adjustment for multiple testing are listed in Supplementary Table S2. For all data, an asterisk (*) indicates significance (*P* < 0.05) compared to buffer-only groups, and N = 4–8 mice per group.

To investigate the role of NOD2 expression by CD11c + APCs in antigen-specific humoral immune responses against LaOVA, longitudinal mucosal and systemic antibody responses against OVA were measured by ELISA. Fecal, serum, and vaginal lavage samples were collected from mice that were gavaged with LaOVA, control *L. acidophilus* strain NCK1895, or dosing buffer alone. These mice either had functional NOD2 (CD11c^cre^) or NOD2 was knocked out in CD11c-expressing cells (NOD2^ΔDC^). Additional mouse groups included two genotype controls, NOD2^fl^-CD11c^cre^ and NOD2^fl/fl^ (Table 1). IgA and IgG OVA-specific antibody responses from each experimental group are shown in Fig. 1a–c. Optical density readings from week 0 were used to determine the cutoff dilutions for endpoint titers. An early OVA-specific IgA humoral immune response was seen in fecal samples for all mice harboring functional NOD2 in DCs and vaccinated LaOVA (*CD11c*^*cre*^ + LaOVA, *NOD2*^*fl*^*-CD11c*^*cre*^ + LaOVA, and *NOD2*^*fl/fl*^ + LaOVA, Fig. 1a). Anti-OVA endpoint titers in these groups were all significantly higher than those in the *NOD2*^*ΔDC*^ groups by week 6 (Fig. 1a). Additionally, significant levels of fecal anti-OVA IgA were detected in *NOD2*^*fl/fl*^ + LaOVA mice at weeks 2 and 4, and *CD11c*^*cre*^ + LaOVA had significant levels at week 4 compared to the NCK1895 and Buffer groups. Significant production of fecal anti-OVA IgA continued in these three groups throughout the end of the study.

**Table 1:** Genotype and treatment of mouse groups used in this study.

Group	*NOD2-Flx*	*Cd11c-Cre*	Purpose	Treatment
*CD11c*^*cre*^ + NCK1895	−/−	+	Cre control	NCK1895
*CD11c*^*cre*^ + LaOVA	−/−	+	Cre control	LaOVA
*CD11c*^*cre*^ + Buffer	−/−	+	Cre control	Buffer
*NOD2*^*ΔDC*^ + NCK1895	+ / +	+	NOD2^DC^-Kockout	NCK1895
*NOD2*^*ΔDC*^ + LaOVA	+ / +	+	NOD2^DC^-Kockout	LaOVA
*NOD2*^*ΔDC*^ + Buffer	+ / +	+	NOD2^DC^-Kockout	Buffer
*NOD2*^*fl*^*-CD11c*^*cre*^ + LaOVA	+ /−	+	Single NOD2-Knockout	LaOVA
*NOD2*^*fl/fl*^ + LaOVA	+ / +	−	NOD2-loxP control	LaOVA

Similarly, serum anti-OVA IgG endpoint titers in the *CD11c*^*cre*^ + LaOVA and two genotypic controls receiving LaOVA (*NOD2*^*fl*^*-CD11c*^*cre*^ + LaOVA and *NOD2*^*fl/fl*^ + LaOVA) all had significant antibody levels starting at week 8 when compared to buffer groups (Fig. 1b) and continued through the end of the study. The genotype control group *NOD2*^*fl/fl*^ + LaOVA also had significantly higher levels of anti-OVA serum IgG after just one dosing series (week 2). Groups *CD11c*^*cre*^ + LaOVA and *NOD2*^*fl*^*-CD11c*^*cre*^ + LaOVA both showed significantly higher levels of vaginal anti-OVA IgA starting at week 6 (Fig. 1c) compared to all other groups. However, the *NOD2*^*fl/fl*^ + LaOVA group did not show significant levels of vaginal anti-OVA IgA and was similar to the titers found in the *NOD2*^*ΔDC*^ + LaOVA mice (Fig. 1c). Conversely, *NOD2*^*fl/fl*^ + LaOVA had significant levels of anti-OVA antibodies in fecal and serum samples after the first vaccination series compared to buffer groups (Fig. 1a,b, week 2). These results show the strong immunogenicity of the LaOVA vaccine construct and the essential role of NOD2 in mounting an antigen-specific immune response for both IgA and IgG.

Total IgA and OVA-specific antibody secreting cells (ASCs) were determined via ELISpot. Lymphocytes were collected from the female reproductive tract (FRT), large intestine (LI), mesenteric lymph nodes (MLN), Peyer’s patches (PP), and spleen (Sp) at sacrifice for both flow cytometry and ELISpot (Fig. 1d,e). Flow cytometry gates were used to obtain live B cell population percentages (7AAD^-^CD45^+^CD19^+^), which were used in the calculation of spot forming units (SFU) for total IgA- and OVA-specific IgA-producing cells. Each tissue that was assessed had a significantly higher number of OVA-specific SFUs from at least one of the LaOVA-dosed groups with functional DC-NOD2 (Fig. 1d). Low numbers of OVA-specific SFUs were detected in the spleen and FRT tissues of *NOD2*^*ΔDC*^ + LaOVA mice but were not significant compared to the buffer and NCK1895 groups. (Fig. 1d). The levels of total IgA-ASCs were similar between all the groups, with the exception of *NOD2*^*fl*^*-CD11c*^*cre*^ + LaOVA, where both the LI and MLN had significantly higher counts than at least one other group (Fig. 1e). P values, adjusted for multiple testing, for each pairwise comparison for both ELISA and ELISpot analyses are shown in Tables S1 and S2. Together, these results highlight the critical role that NOD2 plays specifically in CD11c + APCs in mounting both mucosal and systemic humoral immune responses against LaOVA.

### NOD2 signaling and LaOVA vaccination impacts cytokine production

**Figure 2:**
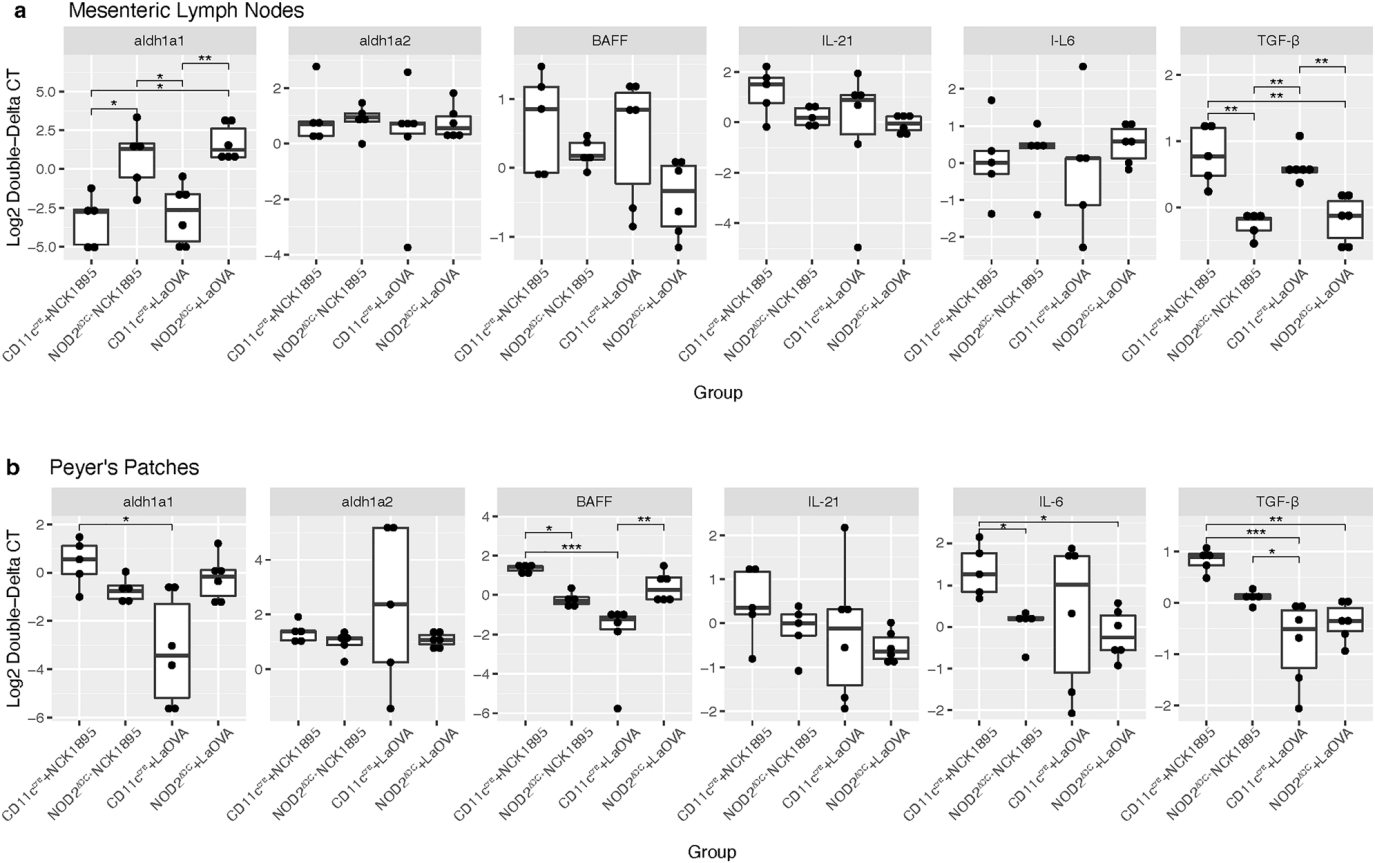
Cytokine production following oral vaccination. Eighteen hours after vaccination, mice were sacrificed, and cells from mesenteric lymph nodes (**a**) and Peyer’s patches (**b**) were saved for analysis of cytokine expression using RT–qPCR. Relative changes in expression were determined using the 2^−ΔΔCT^ method with the buffer group from the respective genotype (CD11c^cre^ or NOD2^ΔDC^) being used for baseline expression levels. Significance is represented by (*) for (*P* < 0.05), (**) for (*P* < 0.01), and (***) for (*P* < 0.001), as determined by the Kruskil-Wallis test of analysis of variance with Dunn’s multiple comparison test and Benjamini–Hochberg adjustment (Supplementary Table S3).

Cytokines important for IgA class switching and secretion were analyzed in both MLN and PP tissues via RT–qPCR. These cytokines included retinaldehyde dehydrogenase 1 (*aladh1a1*), retinaldehyde dehydrogenase 2 (*aladh1a2*), B cell-activating factor (*BAFF*, also known as tumor necrosis factor ligand superfamily member 13B (*Tnsf13b)), IL-6, IL-21*, and transforming growth factor beta (*TGF-β*). To measure the effects of vaccination, cytokines were measured 18 h after the final vaccination during week 12. Levels are reported as log change compared with the buffer group from the respective genotype. Significant differences were found between both retinaldehyde dehydrogenase-1 and *TGF-β* in both tissues, as well as *BAFF* in Peyer’s patches (Fig. 2), indicating that these cytokines are impacted by the NOD2 signaling pathway.

Notably, MLN tissues showed significant differences in retinaldehyde dehydrogenase-1 and TGF-β in all groups except between groups with similar genotypes (Fig. 2a). Retinaldehyde dehydrogenase-1 levels were elevated in the *NOD2*^*ΔDC*^ + LaOVA and slightly elevated in *NOD2*^*ΔDC*^ + NCK1895 mice, while levels were significantly decreased in *CD11C*^*cre*^ + LaOVA and *CD11C*^*cre*^ + NCK1895 mice. Conversely, the levels of *TGF-β* were increased in the *CD11C*^*cre*^ groups and decreased in the *NOD2*^*ΔDC*^ groups in MLN tissues. Similar patterns between the genotypes of mice were seen in *BAFF* and *IL-21* levels but were not significant. PP tissues showed more significant differences based on vaccination strain but only within the *CD11C*^*cre*^ groups (Fig. 2b). Levels of retinaldehyde dehydrogenase-1 (*aldh1a1*) were increased in the *CD11C*^*cre*^ + NCK1895 group and significantly decreased in the *CD11C*^*cre*^ + LaOVA group relative to each other. Similarly, *TGF-β* levels were significantly increased in the *CD11C*^*cre*^ + NCK1895 group compared with the decreased levels found in *CD11C*^*cre*^ + LaOVA and *NOD2*^*ΔDC*^ + LaOVA. Significant differences in *BAFF* levels appeared between *CD11C*^*cre*^ + LaOVA and the increased levels of *NOD2*^*ΔDC*^ + LaOVA and *CD11C*^*cre*^ + NCK1895. *NOD2*^*ΔDC*^ + LaOVA and *CD11C*^*cre*^ + NCK1895 also had significantly different levels of BAFF. The cytokine IL-6 was elevated in both *CD11C*^*cre*^ + NCK1895 and *CD11C*^*cre*^ + LaOVA, with levels in *CD11C*^*cre*^ + NCK1895 being significantly higher than those in the *NOD2*^*ΔDC*^ groups. Collectively, these results show the multiple impacts of both inhibition of NOD2 signaling and administration of *L. acidophilus* on these cytokines with known importance to IgA production.

### NOD2 signaling alters bacterial microbial diversity and composition

Several NOD2 polymorphisms have been directly associated with Crohn’s disease^23^, and mice with NOD2 deficiency often have an altered microbiota resulting from the lack of maintenance provided by NOD2^13,24^. However, the role of NOD2 specifically in dendritic cells in relation to disturbances to the microbiome has yet to be shown. 16S rRNA sequencing of the fecal microbiome was used to better understand this unknown relationship. To investigate potential changes in the abundances of different taxonomic classification of bacteria over time, the relative abundances of OTUs at each timepoint in the experimental groups at the family and phylum level were plotted (Supplementary Fig. S1a and S1b, respectively). The family *Muribaculaceae* had the highest abundance in nearly all timepoints, with *Lactobacillaceae* also having a high prevalence throughout the study (Supplementary Fig. S1b). As expected, Bacteroidetes and Firmicutes had the highest abundance at the phylum level (Supplementary Fig. S1b). Very few proteobacteria were discovered throughout the study and were only detected in *CD11C*^*cre*^ + LaOVA at week 6 and *NOD2*^*ΔDC*^ + NCK1895 at week 10.

**Figure 3:**
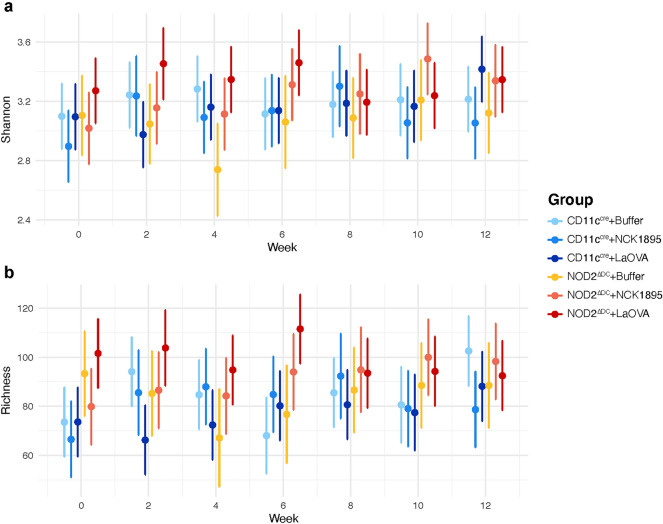
Alpha Diversity. Predicted values of Shannon diversity (**a**) and richness (**b**) at each timepoint (weeks − 2, 0, 2, 4, 6, 8, 10, 12) are represented by the 95% confidence intervals. Linear mixed effects models were used to determine predicted values for each diversity index (Shannon and observed richness). No overlap between the confidence intervals indicates significant difference between in diversity or richness at any time point.

Predicted values of alpha diversity, as shown by both the Shannon index and richness, appear to be influenced by both NOD2 function and vaccination (Fig. 3). Figure 3a shows the initial prevaccination differences in predicted values of richness at week 0 between the different genotypes. These differences expanded after mice were vaccinated. This was especially apparent at week six, where NOD2^ΔDC^ + LaOVA mice had the highest predicted value of richness and was significantly higher than NOD2^ΔDC^ + Buffer, CD11C^cre^ + Buffer, and CD11C^cre^ + LaOVA. While NOD2^ΔDC^ mice generally had higher alpha-diversity, the NOD2^ΔDC^ + Buffer group had the lowest value at week four. A similar trend was observed in Shannon diversity (Fig. 3b), with values for NOD2^ΔDC^ + Buffer being significantly lower than both NOD2^ΔDC^ + LaOVA and CD11C^cre^ + Buffer. Additionally, both predicted richness and Shannon diversity for NOD2^ΔDC^ + LaOVA were significantly higher than CD11C^cre^ + LaOVA values after just one round of vaccination (week 2, Fig. 3a,b). Notably, the predicted values of both richness and Shannon diversity were very similar for weeks 8–12, with no significant differences between groups, indicating that the initial disruption caused by administration of *L. acidophilus* was resolved by week eight. These results provide initial evidence that innate NOD2 signaling in CD11c + DCs impacts the alpha diversity of the bacterial microbiome, but differences due to both NOD2 and repeated administration of *L. acidophilus* are temporary and were not found past the 6th week.

**Figure 4:**
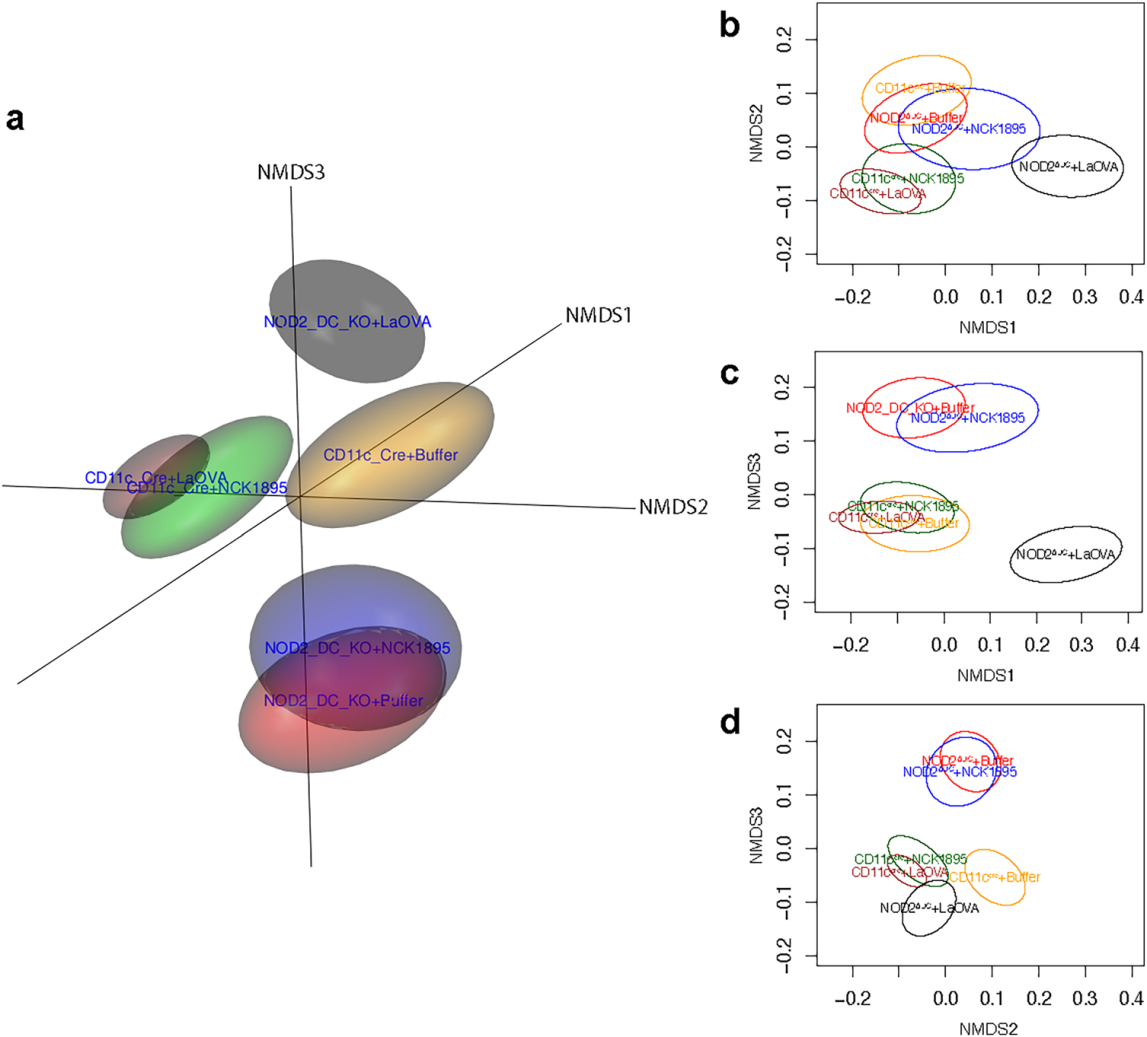
Changes to beta diversity from genotype and *L. acidophilus* administration. Nonmetric multidimensional scaling (NMDS) plots show alterations to the microbiome community structure between groups. (**a**) The three-dimensional plot represents the three NMDS axes. The two-dimensional projections NMDS1 and NMDS2 are shown in (**b**), NMDS1 and NMDS3 are shown in (**c**), and NMDS2 and NMDS3 are shown in (**d**). Data are represented by the 95% confidence ellipsoids and include all samples from all timepoints from each experimental group, where nonoverlapping groups denote significance.

Figure 4 highlights the differences in beta diversity of the microbiome based on both vaccination and the NOD2 genotype. The figure indicates differences in the microbiome between the wild-type CD11C^cre^ and NOD2^ΔDC^ mice in general, where there is no overlap between the ellipsoids associated with these genotypes at any treatment level (Fig. 4a). Response to perturbation due to treatment within the NOD2^ΔDC^ genotype was different compared to the CD11C^cre^ genotype. The CD11C^cre^ groups separated based on administration of *L. acidophilus*; CD11C^cre^ + LaOVA and CD11C^cre^ + NCK1895 groups are not significantly different from each other but are both significantly different compared to the negative control Buffer group (Fig. 4b). Alternatively, the NOD2^ΔDC^ + LaOVA group was significantly different than both the NCK1895 and Buffer NOD2^ΔDC^ groups. The combined effect of CD11c-NOD2 signaling and LaOVA vaccination on the microbiome was further shown by 2D NMDS projections (Fig. 4b,c). These results highlight the increased susceptibility to disturbances to the microbiome in mice with NOD2^ΔDC^ from LaOVA vaccination compared to wild-type CD11C^cre^ mice.

**Figure 5:**
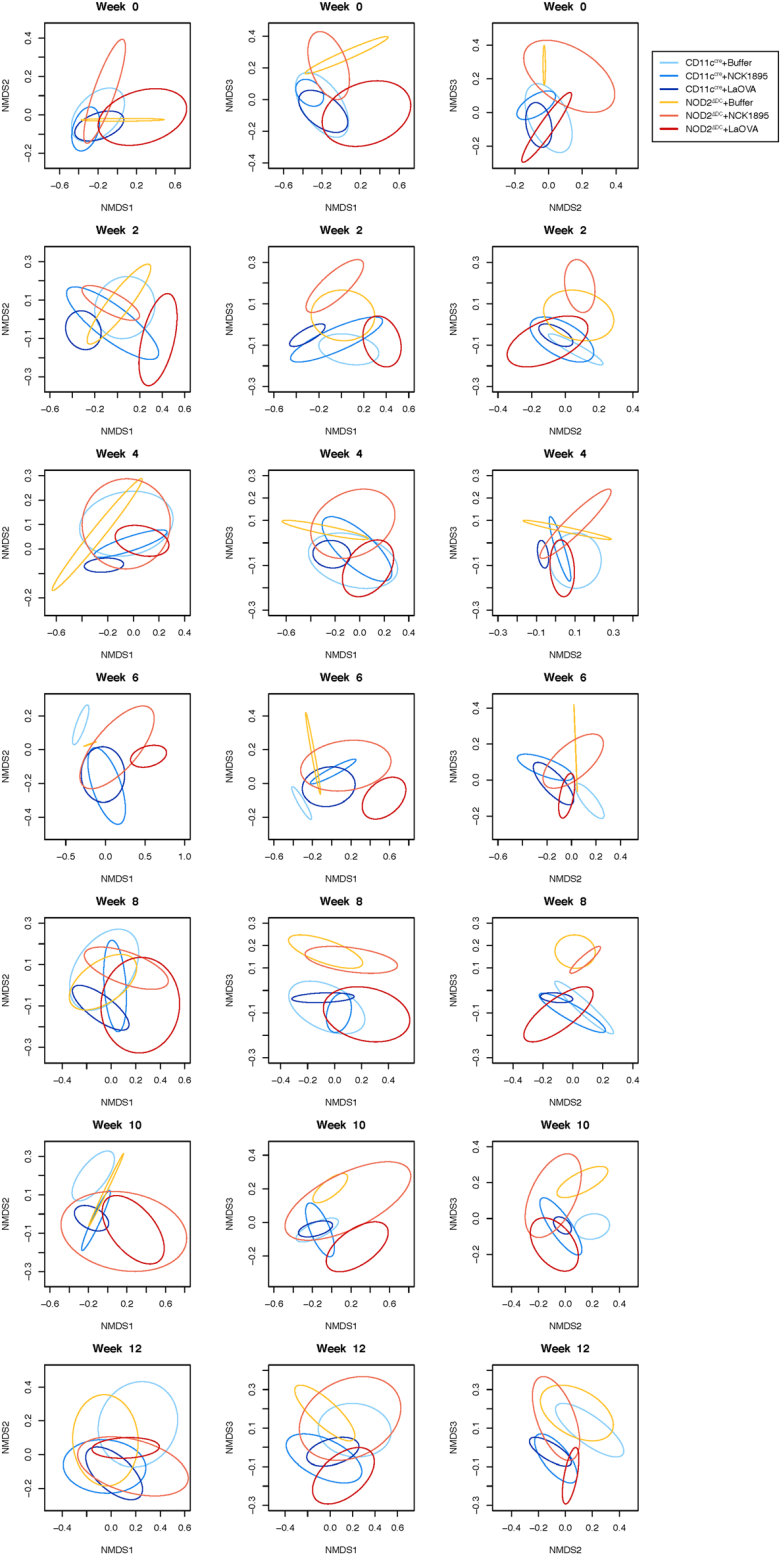
Temporal changes in beta-diversity. NMDS ordinations with data separated into plots for each timepoint (week). The three columns show the projections of NMDS1, NMDS2, and NMDS3, with NMDS1 and NMDS2 in column 1, NMDS1 and NMDS3 in column 2, and NMDS2 and NMDS3 in column 3. Ellipsoids represent the 95% confidence intervals for each group at each timepoint.

To understand the temporal changes in the microbiome after repeated oral boosters of *L. acidophilus*, NMDS plots were generated to show the microbial composition of each group at each timepoint (Fig. 5). The 2D projections of the 3D ordination are shown in each column, with NMDS1 and NMDS2 in column 1, NMDS1 and NMDS3 in column 2, and NMDS2 and NMDS3 in column 3. The prevaccination microbiome is shown in the first plots (week 0), indicating an overlap between all ellipsoids as expected prior to *L. acidophilus* administration. An immediate alteration after one round of dosing with LaOVA is seen in the week 2 plot by the separation between the NOD2^ΔDC^ + LaOVA and NOD2^ΔDC^ + NCK1895. Differences in microbiome composition were greatest at week 6, with the least amount of overlap between groups occurring at this time point. Separation between the CD11C^cre^ groups and the NOD2^ΔDC^ + Buffer and NOD2^ΔDC^ + NCK1895 animals was most prominent at week 8 and week 10 for NOD2^ΔDC^ + Buffer mice. The expanded ellipsoids for both the NOD2^ΔDC^ + NCK1895 and NOD2^ΔDC^ + LaOVA groups during week 10 also signifies a greater variance between NOD2^ΔDC^ animals administered *L. acidophilus*, alluding to NOD2’s role in stabilizing the microbiome during vaccination*.* Temporal changes within each group can also be seen in Figure S2, where NMDS plots show the similarities of the microbiome within each experimental group throughout the study with no indication of a major shift in the microbiome state from the starting point. Week 6 (represented by gray ellipses in each group) again shows the most differences, especially within the NOD2^ΔDC^ + LaOVA and CD11C^cre^ + Buffer plots. Between the two genotypes, the microbiome of the NOD2^ΔDC^ groups appeared to be less stable than that of the CD11C^cre^ animals (Figure S2), as indicated by the greater shifts between the centroids of each ellipsoid. Together, these results provide evidence for the critical role of NOD2 signaling in the composition of the intestinal microbiome when a live probiotic is administered.

### Random forests reveals important taxa for classification

To identify taxa that were important for the separation of experimental groups (treatment and genotype), the Random Forests^25^ (RF) machine learning approach was used. The RF model used the experimental group (Table 1) as the target of classification and identified operational taxonomic unit (OTU) drivers of classification. The confusion matrix shows the respective error rate and classification for each group (Table 2). Overall, the RF model had a 13.49% out-of-bag (OOB) classification error rate with an overall range between 4.88% and 24.24%. The highest per-group error rate belonging to the CD11C^cre^ + NCK1895 group (24.24%) where out of the 33 CD11C^cre^ + NCK1895 samples, 25 were correctly assigned and 7 were erroneously classified as CD11C^cre^ + LaOVA, again indicating that these two groups had similar microbiomes. This pattern was not observed in the NOD2^ΔDC^ groups, with misclassification error rates ranging between 4.88% and 15.38%. For all classifications, error rates were minimized by selecting the optimal number of features to use when creating the regression trees for the RF model. The lowest median OOB error was 25 and is shown in Supplementary Figure S3.

**Table 2:** Confusion Matrix.

	CD11c^cre^ + Buffer	CD11c^cre^ + NCK1895	CD11c^cre^ + LaOVA	NOD2^ΔDC^ + Buffer	NOD2^ΔDC^ + NCK1895	NOD2^ΔDC^ + LaOVA	Class error
CD11c^cre^ + Buffer	35	0	3	0	1	1	0.125000
CD11c^cre^ + NCK1895	0	25	7	0	1	0	0.242424
CD11c^cre^ + LaOVA	2	2	36	0	0	1	0.121951
NOD2^ΔDC^ + Buffer	0	2	1	22	1	0	0.153846
NOD2^ΔDC^ + NCK1895	3	1	0	0	29	1	0.147058
NOD2^ΔDC^ + LaOVA	0	0	2	0	0	39	0.048780

Type of Random Forests: classification.

Number of trees: 1000.

Number of variables tried at each split: 25.

OOB estimate of error rate: 13.49%.

**Figure 6:**
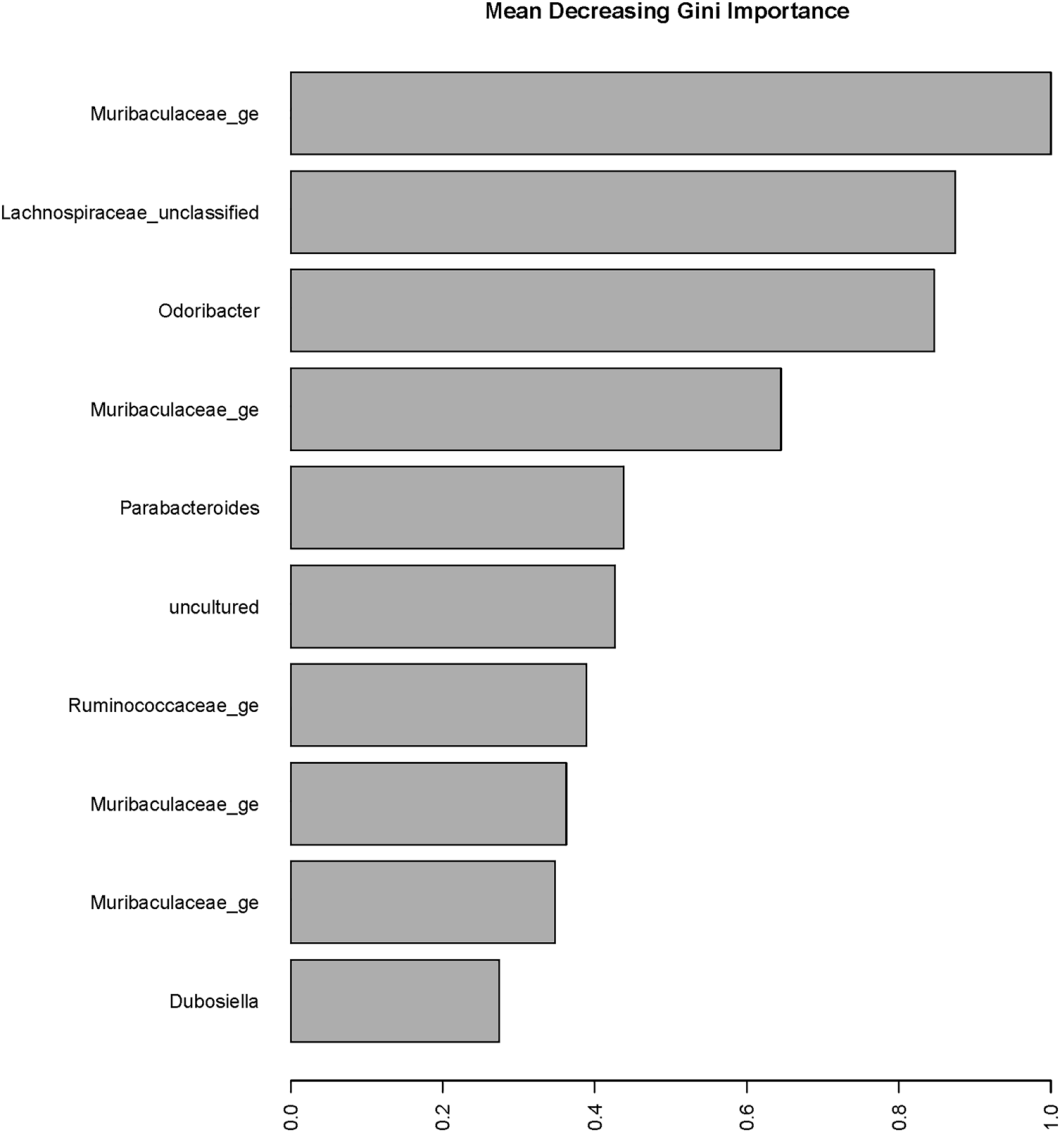
Mean decreasing Gini (MDG) coefficient plot for important OTUs. The top 10 important OTUs, as identified from the Random Forests’ classification for experimental groups, are displayed by the associated genus and in order of MDG coefficient.

Figure 6 identifies the taxa that were classified as most important, represented by the Mean Decreasing Gini (MDG) coefficient. The genus *Muribaculaceae_ge* was recognized as the most important driver, and three additional OTUs in the same genus were among the top 10 important taxa. *Odoribacter* was identified as the third most important OTU for classification (Fig. 6). The other Bacteroidetes OTU that was identified was OTU0147 and is a member of the *Parabacteroides* genus. Other OTUs identified with the highest MDG included members of the Firmicutes phylum, including *Lachnospiraceae_unclassified*, an uncultured genus in the Lachnospiraceae family, *Ruminococcaceae_ge*, and *Dubosiella*. The complete list of OTUs with associated MDG coefficients is shown in Supplementary Table S4.

**Figure 7:**
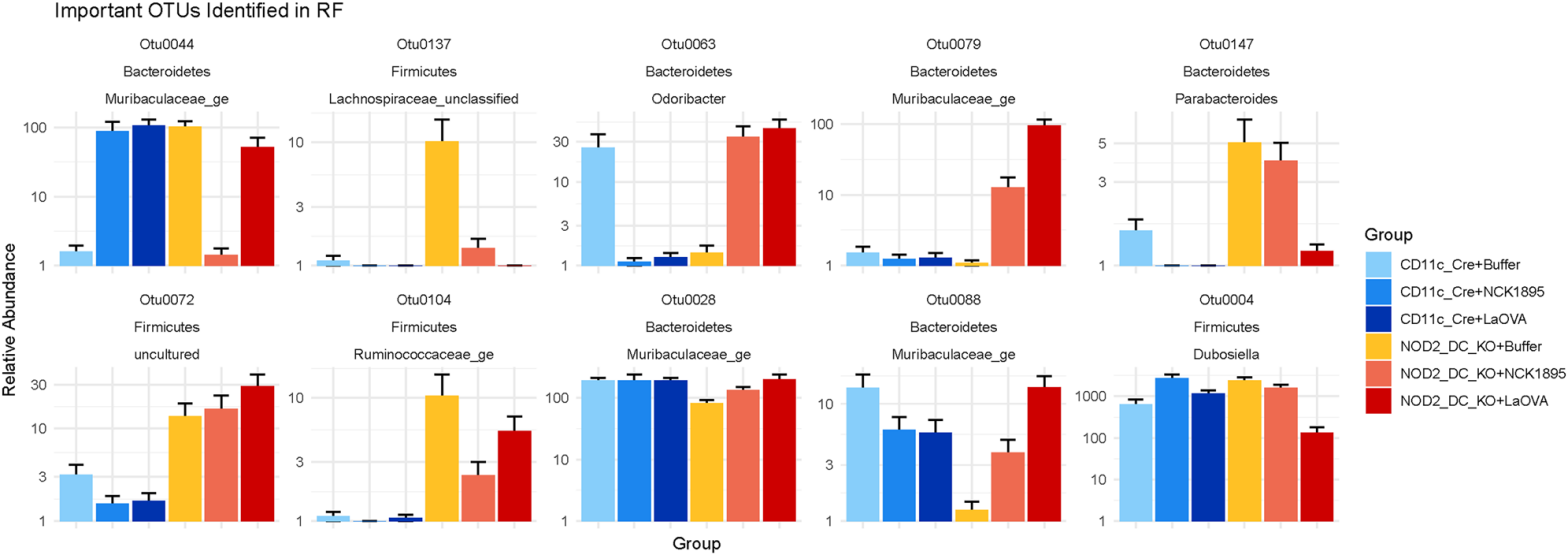
Relative abundance of important taxa. The relative abundances of the top ten important taxa identified in the Random Forests’ classification are shown. The phylum and genus classification for each OTU is also displayed.

To further understand why these OTUs were highlighted as important in the RF model, the abundances of each OTU were plotted. Figure 7 displays the relative abundances within each experimental group for the top 10 OTUs that were identified by the RF model in order of MDG. The phylum and genus of each OTU are displayed above the individual plots. Two Firmicute members, OTU0104 (*Ruminococcaceae_ge*) and OTU0072 (uncultured genus in the Lachnospiraceae family), had higher abundances within the NOD2^ΔDC^ groups. Each of the four OTUs identified within the *Muribaculaceae_ge* genus were all highly abundant in the NOD2^ΔDC^ + LaOVA group. The only Bacteroidetes that was not highly abundant within the NOD2^ΔDC^ + LaOVA group was OTU0147, which belonged to the *Parabacteroides* genus. A NCBI BLASTN search using consensus fastq files from these OTUs was conducted to obtain further taxonomic classifications. OTU0063 aligned with *Culturomica sp.*, a bacterium that has recently been isolated from the human gut. The strain of *Culturomica massiliensis* was 91% similar to *Odoribacter laneus*, allowing its new genus classification. OTU0147, originally classified in the Parabacteroides genus, was further identified as a *P. goldsteinii* species. Collectively, these RF results show that the composition of the microbiome was highly influenced by both the genotype and treatment, as indicated by the low error rates between all experimental groups. Additionally, the *Muribaculaceae* genus was responsible for many of the correct classifications due to many OTUs with a high MDG coefficient (Supplementary Table S4).

## Discussion

NOD2 signaling, specifically in CD11c + cells, was assessed to understand NOD2’s role in mounting an antigen-specific humoral immune response against our recombinant *Lactobacillus acidophilus* vaccine platform. Using mice with CD11c-specific NOD2 knockout, we demonstrated that NOD2 signaling in CD11c + phagocytic cells is required for the stimulation of the OVA-specific humoral immune response both in the mucosa and systemically based on antibody responses from mucosal sites and serum (Fig. 1). Cytokine profiles provided additional support for the role of NOD2 in the humoral response to rLA, as several important cytokines for IgA class switching were not upregulated in CD11c-NOD2-deficient mice (NOD2^ΔDC^ groups, Fig. 2). The results presented here also indicated that NOD2 plays an important role in the maintenance of the microbiome in response to administration of a live probiotic. All experimental groups were identified with relatively high accuracy with RF, indicating that genotype and *L. acidophilus* administration both influence the microbiome composition. Importantly, the microbiome composition was primarily separated based on genotype and secondarily based on administration of either LaOVA or NCK1895 (Fig. 4).

Signaling from innate pattern recognition receptors is critical for mounting an adaptive immune response, and NOD2 has long been exploited by Freund’s adjuvant containing MDP from Mycobacterium^26^. More recently, novel adjuvants and muramyl peptide derivatives targeting NOD2 have shown great potential at further increasing the immunogenicity of current vaccine constructs, including BCG, the nanoparticulate HIV-1 p24 vaccine, and the influenza subunit vaccine, among others^27–29^. Since it is also known that the structure of MDP can result in varying stimulation of NOD2 based on its acylation, we previously demonstrated that *L. acidophilus* was capable of activating NOD2 in vitro^21^. That study also recognized NOD2 as a key factor in the humoral immune response against rLA in vivo, but specific cell types remained unidentified. Here, we provided evidence that NOD2 signaling specifically in CD11c + APCs, such as dendritic cells, is critical for the humoral immune response against LaOVA.

NOD2 played a role in both the mucosal immune response and trafficking to the systemic immune system throughout vaccination with LaOVA (Fig. 1). Furthermore, cytokine responses corroborated the absence of a humoral immune response in the *NOD2*^*ΔDC*^ groups (Fig. 2). TGF-β plays a dual role in regulating the survival and proliferation of B cells by inducing apoptosis and growth arrest and promoting the production of IgA through IgA class switching^30^. The increase in this cytokine in the MLN of *CD11C*^*cre*^ mice administered strains of *L. acidophilus* (LaOVA and NCK1895) indicates that the humoral immune response is coordinated through TGF-β signaling while promoting the production of OVA-specific IgA. The low levels of *TGF-β* in both the MLN and PP of NOD2^ΔDC^ animals further confirm that deficient NOD2 signaling in CD11c + DCs inhibits the humoral immune response that is controlled by TGF-β signaling. However, the results from Peyer’s patches were less definitive, with the exception of IL-6. This cytokine has previously been shown to be an important regulator of IgA production in response to the LAB *Pediococcus acidilactici* K15^31^, and the data shown here indicate that *L. acidophilus* is also a potent inducer of IL-6 in PPs. The lack of *IL-6* production in NOD2^ΔDC^ groups also provides insight into the downstream effects of NOD2 signaling in CD11c + DCs in response to the commensal microbiota.

NOD2 is of particular interest in relation to inflammatory bowel diseases, especially Crohn’s disease (CD). Although the specific etiology of CD is still unknown and likely multifactorial, meta-analyses of CD cohorts have revealed that specific mutations in *NOD2* are strongly associated with CD onset, with the majority being ileal CD, as well as other inflammatory bowel diseases^32–36^. One characteristic of CD is a shift in the composition of the microbiome, usually with an increase in pro-inflammatory *Proteobacteria* species^37^. The resulting dysbiosis connected to NOD2 was thought to be related to a decrease in antimicrobial peptide production from Paneth cells and thus assumed to be a result of NOD2 mutations in nonhematopoietic cells^38^. However, the results presented here demonstrate that NOD2 signaling in antigen-presenting cells of hematopoietic origin (CD11c +) also plays a critical role in maintaining the composition of the baseline microbiome, specifically shown by the separation of the microbiome composition between NOD2^ΔDC^ and CD11c^cre^ mice belonging to the same treatment group (Fig. 4). Furthermore, the vast separation of NOD2^ΔDC^ + LaOVA compared to NOD2^ΔDC^ + NCK1895 and NOD2^ΔDC^ + Buffer in Fig. 4 indicates that NOD2 signaling in CD11c + is required to prevent a shift in community structure during an innate immune response associated with LaOVA. The lack of anti-OVA antibodies in NOD2^ΔDC^ suggests that only an innate response was initiated during vaccination, and LaOVA vaccination was enough to alter the microbiome without regulation of NOD2 signaling. Conversely, the similarities between the CD11c^cre^ mice administered LaOVA or NCK1895 suggest that the impact on the microbiome by these strains was comparably regulated by NOD2 signaling. Figure 5 shows that these two groups had similar microbiome compositions throughout the study, with the exception of week 4.

The results from random forest indicated that several taxa were instrumental in classifying experimental groups correctly, with many taxa belonging to the *Muribaculaceae* genus. In addition to *Muribaculaceae,* other Bacteroidetes genera with high MDG coefficients included *Odoribacter* and *Parabacteroides*. All of these genera are common commensals of the mammalian gut microbiome, with decreased abundances of *Odoribacter* specifically linked to inflammatory bowel disease (IBD)^39^. Recent results have shown that *Muribaculaceae* is negatively correlated with inflammation in a colitis mouse model^40^. Although this genus was often abundant in the NOD2^ΔDC^ groups, the species and strain differences within this and many other genera should be considered. For this reason, an NCBI BLAST search was conducted to further classify important OTUs at either the species or strain level. OTU147 was aligned with *Parabacteroides goldsteinii*, which has been shown to reduce obesity in mice fed a high-fat diet^41^. Additionally, OTU063 aligned to *Culturomica sp.*, a newly isolated bacterium from the human gut^42^, and is phylogenetically close to *Odoribacter* (OTU063 was classified based on our alignment). However, the function of this species remains unknown.

Taken together, the results presented here show that humoral immune induction by the rLA platform is restricted to the NOD2 pathway with no evidence of redundancy through other innate immune pathways. This is in contrast to bacterial pathogens such as Salmonella, where a robust immune response was achieved with both MYD88/TRIF- and Caspase-1/-11-deficient mice^43^. Further studies are needed to identify compartments of the microbiome that may be disproportionately affected by a dysfunction in NOD2 along the gastrointestinal tract, especially within the small intestine, where NOD2 is thought to be most active^24,32^. Future mucosal vaccine development will require targeting critical immune pathways in conjunction with nutritional priming of the gastrointestinal tract to achieve high efficacy globally.

## Materials and methods

### Ethics statement and study design

This study was carried out under strict accordance with recommendations in the Guide for the Care and Use of Laboratory Animals of the National Institutes of Health and ARRIVE guidelines (https://arriveguidelines.org). Protocol 17-7495A was approved by the Colorado State University Institutional Animal Care and Use Committee (IACUC). Animal welfare and health were monitored daily, and in instances where medical intervention was not effective, animals were humanely euthanized, and every effort was made to minimize animal suffering.

**Figure 8:**
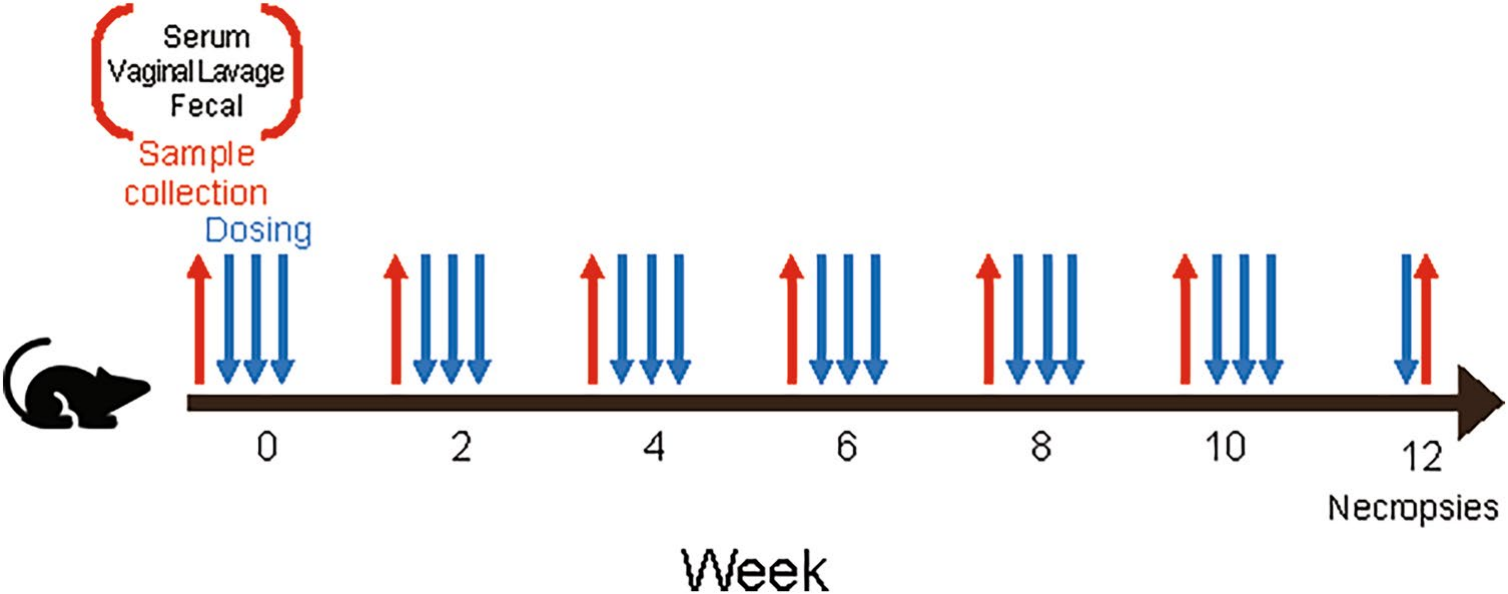
Experimental design and vaccination schedule. Samples were collected from mice every two weeks, with vaccination beginning immediately after collection. Mice were immunized with buffer only, NCK1895, or LaOVA once every three days at each boost.

Four to eight mice were assigned to experimental groups (described in Table 1), depending on the availability of mice with the correct genotype for each group during breeding for a total of 45 mice. These numbers of animals per group were determined by power calculation in R using ELISA results from previous studies. Vaccines were delivered intragastrically three days in a row during weeks 0, 2, 4, 6, 8, and 10, with an additional dose 18 h before sacrifice at week 12 for stimulation of cytokine production (Fig. 8, and Supplementary Materials and Methods). Mice were housed in groups of two to four. Two weeks after the last dosing timepoint, mice were euthanized, and tissues were processed to obtain single-cell suspensions (Supplementary Materials and Methods). Blood, fecal, and vaginal samples (315 samples for each) were collected from each animal prior to administration of vaccination for investigation of antibody titers (Supplementary Materials and Methods) for ELISAs. Additionally, 315 samples were collected for microbiome analysis throughout the study. At the end of the study, 90 samples between mesenteric lymph nodes and Peyer’s patches were saved for cytokine analysis, and 366 samples were processed for ELISpot analysis. Throughout analysis, the dosing buffer groups were used as the negative control to compare the LaOVA and NCK1895 results, and the randomization strategy for library preparation is described in the microbiome methods below.

### Bacterial culture conditions

*Lactobacillus acidophilus* recombinant strains LaOVA and NCK1909 were grown in MRS broth (BD Diagnostics, Sparks, MD), and strain NCK1895 was grown in MRS with 5 μg/ml erythromycin (Em)^10^. *Lactobacillus* cultures were incubated overnight at 37 °C under static conditions. *Escherichia coli* strains were grown aerobically with shaking in LB medium (BD Diagnostics) with or without 200 μg/ml Em and 40 μg/ml kanamycin (km) at 37 °C.

### Construction of rLA-OVA and verification of OVA expression

Recombinant *L. acidophilus* expressing the peptide OVA_323-339_ (LaOVA) on the surface layer protein A (slpA) was generated using methods similar to those previously described^10,44^ (also described in the Supplementary Materials and Methods).

### Tissue-specific NOD2 knockout mice

NOD2-floxed (NOD2^fl/fl^) mice and CD11c-Cre mice were bred to generate mice with a tissue-specific knockout of NOD2 used in this study. NOD2^fl/fl^ mice on the C57BL/6 background were provided by Dr. David Prescott at the University of Toronto^18,45^ (described in Supplementary Materials and Methods).

One concern with any animal study using DNA nickases for gene editing is the potential for nonspecific side effects on the normal immune response. Mice expressing Cre (CD11c^cre^) were compared with *NOD2*^*ΔDC*^ groups so that any genotoxic effects induced by Cre were similar between all mice. Extra genotype controls were also designed to ensure that loxP sites did not interfere with NOD2 function. Therefore, we are confident that the results presented here are due to the critical role of NOD2 and not the off-target effects of Cre recombinase. All mice were kept under specific pathogen-free conditions and had ad libitum water and standard chow at CSU’s Lab Animal Resources (LAR) throughout the duration of the study.

### Colorimetric ELISA and ELISpot assay

An enzyme-linked immunosorbent assay (ELISA) was developed for the detection of OVA-specific murine antibodies from serum, fecal, and vaginal samples (see Supplementary Materials and Methods).

### RT–qPCR cytokine analysis

Cells collected from MLNs and PP at the end of the study were used to analyze the mRNA expression of several cytokine targets, as further described in the Supplementary Materials and Methods.

### Statistical analysis of immunological data

ELISA, ELISpot, and cytokine data were analyzed using R version 3.6.1. Since these data were not normally distributed, a Kruskil-Wallis test of analysis of variance was used. Dunn’s multiple comparison test was performed post hoc, with Benjamini–Hochberg adjustment applied for correction of multiple comparisons. Statistical significance was set to *P* < 0.05 for all data, with adjusted p values being used. Adjusted P values for pairwise comparisons from ELISA, ELISpot, and cytokine data are shown in Tables S1-S3.

### Microbiome library preparation

Murine fecal pellets collected from animals were stored at -80 °C until all samples could be processed. Randomization was used to minimize any batch-bias. Samples were organized into a 96-well plate format, where temporal samples from each animal were all processed on the same plate, and animals from each group were randomized between the four plates. This method of randomization reduces within mouse variability by removing plate-to-plate bias that may contaminate temporal analysis. Each plate received a set of controls, which included no-template controls (NTCs) from extractions and PCR to track any possible introduction of contamination. Several microbial community standards (mock communities) from ZymoBIOMICS were used as positive controls to assess DNA extraction efficiency and PCR errors from different microbial taxa. Both the ZymoBIOMICS Gut Microbiome Standard (D6331) and ZymoBIOMICS Microbial Community standard (D6300) were used as positive controls for DNA extraction, and the ZymoBIOMICS Microbial Community DNA Standard (D6305) was used as a positive control for PCRs. DNA was extracted from all samples using the ZymoBIOMICS 96 DNA Kit, utilizing lysis tubes to prevent sample-to-sample contamination during the lysis of cells. Extracted DNA was used to create Illumina library molecules from the hypervariable region 4 (V4) of the 16S rDNA gene as described previously^9^. Dual indexed libraries were purified using magnetic Mag-Bind TotalPure NGS beads (Omega Bio-Tek), and purified libraries were estimated using the AccuBlue dsDNA Quantitation Kit (Biotim). An equimolar amount of each sample was combined into one pool and sequenced on an Illumina MiSeq with the v2 500-cycle kit at Colorado State University’s Next Generation Sequencing Core Facility (Fort Collins, CO). Details of microbiome data processing and alpha, beta and random forest analyses are described in the Supplementary Materials and Methods.

## Supplementary Information

Supplementary Information 1.

Supplementary Information 2.

## Data Availability

Raw reads associated the microbiome 16S rRNA sequencing are available on the National Center for Biotechnology Information’s (NCBI) Sequence Read Archive (SRA) under BioProject PRJNA751895. All other data are provided in the supplementary tables within this current submission.
