# Supplementary material for: NOD2 signaling in CD11c + cells is critical for humoral immune responses during oral vaccination and maintaining the gut microbiome
Source: Sci Rep. 2022 May 19;12:8491. doi: 10.1038/s41598-022-12469-x (PMC9119386; doi:10.1038/s41598-022-12469-x)
Supplement: Supplementary file 1 — Supplementary Information 1. [file 41598_2022_12469_MOESM1_ESM.pdf]

NOD2 signaling in CD11c+ cells is critical for humoral immune responses during oral vaccination and maintaining the gut microbiome

Bridget E. Fox[1,*], Allison Vilander[1], Zaid Abdo[1,*] and Gregg A. Dean[1,*]

[1]Department of Microbiology, Immunology, and Pathology, Colorado State University, Fort Collins, Colorado 80523, USA

*Corresponding Authors

Supplementary Materials and Methods

Construction of rLA-OVA and Verification of OVA Expression

Recombinant *L. acidophilus* expressing the peptide $OVA_{323-339}$ (LaOVA) on the surface layer protein A (slpA) was generated using methods similar to those previously described[1, 2]. Briefly, the chicken egg Ovalbumin peptide 323-339 was used as a model peptide because it is a known epitope for the I-A(d) major histocompatibility complex class II (MHC-class II) protein, with the sequence ISQAVHAAHAEINEAGR. Plasmid pGAD18 was created using pTRK935 with modified slpA inserted with $OVA_{323-339}$, using published methods[1, 3]. The resulting plasmid was transformed into *L. acidophilus* strain NCK1909. $OVA_{323-339}$ was introduced into the genome via homologous double cross over with the pGAD18. The chromosomal insertion of $OVA_{323-339}$ into *L. acidophilus* was confirmed with both flow cytometry and Sanger sequencing (Genewiz LLC.) for external expression and detection of any mutations, with results shown in Supplementary Figure S5. For flow cytometry, bacterial cells were grown overnight, washed three times with PBS, and incubated with rabbit anti-chicken OVA 323-339 IgG (Alpha Diagnostic Intl. Inc., OVA3231-A) at 10 μg/ml in 1% BSA PBS buffer. Cells were washed and incubated with donkey anti-rabbit IgG conjugated with FITC at 5 μg/ml (Biolegend, San Diego, CA, 406403). Cell population data were collected with the Beckman Coulter Gallios Flow Cytometer and analyzed with FlowJo software. Cells were gated on forward scatter (FSC) and side scatter (SSC) to eliminate debris, and then FL1 was used to identify FITC positive events to indicate OVA-positive expressing cells (Supplementary Figure S5).

Mouse Immunization, Housing, and Sample Collection

Female mice breeding strategies described above were used when they reached 6-8 weeks of age. Live-bacterial vaccines were prepared using freshly grown overnight bacterial cultures. NCK1895 and LaOVA bacterial cells were washed twice in PBS (Corning, Corning, NY) and resuspended in a dosing buffer containing soybean trypsin inhibitor (STI, Sigma) and

sodium bicarbonate ($NaHCO_3$). Mice were given $2x10^9$ CFU of either NCK1895 or LaOVA in 200ul of dosing buffer, or 200 ul of the dosing buffer alone (negative control). Vaccines were delivered intragastrically three days in a row during weeks 0, 2, 4, 6, 8, and 10, with an additional dose 18 hours before sacrifice at week 12 for stimulation of cytokine production. Mice were housed in groups of two to four. Groups with respective genetic background and vaccine treatment are shown in Table 1, and vaccine timeline is shown in Figure 8. Two weeks after the last dosing timepoint, mice were euthanized, and tissues were processed to obtain single-cell suspensions, as described below.

Blood, fecal, and vaginal samples were collected from each animal prior to administration of vaccination for investigation of antibody titers. Fecal samples used for antibody detection were collected and homogenized with PBS supplemented with ProteaseArrest at a 10x weight to volume ratio. Homogenates were spun at 10,000 rpm for 10 minutes to pellet particulates and bacteria. Clear supernatants were aliquoted and stored at -80°C for long term storage. Fecal samples for microbiome analysis were collected directly from the anus of the animal into a sterile PCR tube and placed immediately on ice and transferred to -80°C freezer for long term storage. Serum samples were collected via tail bleeds. Blood was collected with a microvette (Sarstedt, Nümbrecht, Germany) and processed according to manufacturer's protocols for serum isolation. Serum was aliquoted and stored at -80°C. Vaginal lavage samples were collected by gently washing the vagina of mice with 100ul of PBS. The collected fluid sample was immediately put on ice. Samples were then spun at 10,000 rmp *(convert to G)*, and supernatants were aliquoted and stored at -80°C.

Preparation of Single Cell Suspensions

Two weeks after the last immunization, mice were euthanized via carbon dioxide inhalation and thoracotomy. Tissues collected included the spleen (Sp), mesenteric lymph nodes (MLN), Peyer's patches (PP), large intestine (LI), and female reproductive tract (FRT), as

previously described[4, 5]. Briefly, Sp and PP were prepared using a GentleMACS dissociator and filtered through cell strainers to obtain single cell suspensions. The MLNs were isolated and mashed through a cell strainer, washed, and filtered for a single cell suspension. Mucus and epithelium were removed from LI and FRT and placed in digestion media with agitation for 30 minutes at 37°C. Lymphocytes were isolated using a Percol (GE Healthcare) underlay step, then washed and filtered once more to obtained single cells. Viability and concentration of cells were determined using the Cellometer Auto 2000 Cell Viability Counter (Nexcelom Biosciences). Purity of B cells was determined using flow cytometry. Cells were stained with anti-mouse CD45-FITC, CD19-Pacific Blue, and 7-ADD, and gated based on single cells, live cells, CD45+, and CD19+ to obtain antibody-secreting cell (ASC) populations.

Tissue-Specific NOD2 Knockout Mice

NOD2-floxed (NOD2$^{fl/fl}$) mice and CD11c-Cre mice were bred to generate mice with a tissue specific knockout of NOD2 used in this study. NOD2$^{fl/fl}$ mice on the C57BL/6 background were provided by Dr. David Prescott at the University of Toronto[6, 7]. NOD2$^{fl/fl}$ have a proximal loxP site within intron 1 of the *Nod2* gene and the distal loxP site within intron 3. Mice expressing the Cd11c-Cre transgene (Itgax-Cre) on the C57BL/6 background were obtained from the Jackson Laboratory (Bar Harbor, Maine). These two strains of mice were bred to generate NOD2$^{fl/fl}$-CD11c^{cre} mice (referenced as NOD2$^{\Delta DC}$ here). Expression of Cre-recombinase in these NOD2$^{fl/fl}$-CD11c^{cre} mice results in the deletion of the genomic region between the two loxP sites, which includes exon 2 and the cryptic start codon of exon 3, leaving NOD2 non-functional in CD11c+ cells. Genetic controls used in this study included mice heterozygous for the NOD2 loxP sites and expressing Cre-recombinase (NOD2^{fl}-CD11c^{cre}), homozygous for NOD2 loxP sites without Cre-recombinase (NOD2$^{fl/fl}$), and mice expressing Cre-recombinase without loxP sites (CD11c^{cre}). The genotypes of mice were confirmed using PCR to amplify NOD2-lox sites, Cre-recombinase, and an internal Cre control. Primers for

NOD2 included F: 5'-CGGTTGGTGGGATTTCCTGTGC-3' and reverse: 5'-CAGCCAGGGGTGATGATAACAGG-3', which produced a 379-bp band for loxP-negative alleles, and a 499-bp band for alleles harboring the loxP sites. To identify the presence of Cre-recombinase, the following primers were used: Cre transgene F: 5'-CCATCTGCCACCAGCCAG-3', R: 5'-TCGCCATCTTCCAGCAGG-3'; internal Cre control F: 5'-ACTGGGATCTTCGAACTCTTTGGAC-3', R: 5'-GATGTTGGGGCACTGCTCATTCACC-3'. These primers produce a 281-bp band if Cre-recombinase is present and no band if it is absent, and the internal Cre controls primers serve as a positive control which produces a 420-bp band.

Colorimetric ELISA and ELISpot Assay

An enzyme-linked immunosorbent assay (ELISA) was developed for the detection of OVA-specific murine antibodies from serum, fecal, and vaginal samples. Plates (Maxisorp; Nunc, Rochester, NY) were coated with $OVA_{323-339}$ peptide (AnaSpec, Inc., Fremont, CA) at 1 µg/ml in PBS and incubated overnight at 4°C. Plates were washed five times with PBS containing 0.05% Tween-20 (PBST) and blocked with 1% bovine serum albumin (BSA) in PBS for one hour at room temperature (RT). Plates were washed five times again with PBST. Samples were serially diluted in 1% BSA, 0.1% Kathon in PBS and incubated for 2 hours at RT. Plates were washed five times with PBST and incubated with either anti-mouse IgG (Cell Signaling Technology, 20ng/mL) for serum samples, or IgA (Bethyl Laboratories, 40ng/mL) for vaginal wash and fecal samples. Both anti-mouse IgG and IgA antibodies were conjugated with horseradish peroxidase (HRP) and incubated for 1 hour at RT. Plates were washed four times with PBST and three times with PBS. 3,3',5,5'-Tetramethylbenzidine (TMB) peroxidase (SeraCar, Milford, MA) was filtered with a 40uM syringe-filter and acclimated to RT before adding to each well. The reaction was stopped with an equal volume of 1N sulfuric acid. The absorbance was read with a plate reader (BioTek, Winooski, VT), with both 450nm and 570nm recorded (to remove any background noise with 570nm reading).

IgA secreting cells and OVA-specific IgA secreting cells were quantified using the enzyme-linked immunosorbent spot (ELISpot) assay, similar to what has been described previously[2]. Ninety-six-well MultiScreenHTS IP filter plates (Millipore Sigma) were treated with 35% ethanol and washed with sterile distilled water. Plates were coated with 15 µg/ml anti-mouse IgA (Mabtech) in PBS and incubated overnight at 4°C. Plates were washed five times with PBS and blocked with CTL medium for 1 hour at 37°C. Cells from single cell suspensions were added in triplicate at a concentration of 2 X 10^5 for OVA-specific detection and 1 X 10^4 for total IgA. Plates were incubated for 20 hours at 37°C. Plates were then washed with PBST six times to remove cells. For total IgA, 1 µg/ml of biotinylated polyclonal goat anti-mouse IgA (Mabtech) in PBS with 1% FBS was added to each well. For OVA-specific IgA, 1 µg/ml of biotinylated $OVA_{323-339}$ peptide (AnaSpec) was used in the same buffer. Plates were incubated for 2 hours at RT and washed six times. Streptavidin conjugated with horseradish peroxidase (HRP) was added to wells in PBS with 1% FBS and incubated for 1 hour at RT. Plates were washed three times with PBST, and three times with PBS. TMB was filtered with 0.44µM filter and added to wells for either two minutes for total IgA or 10 minutes for OVA-specific IgA. Plates were washed with distilled water ten times and air dried. Spots were counted with an ImmunoSpot analyzer (Cellular Technology Limited).

RT-qPCR Cytokine Analysis

Cells collected from MLN and PP at the end of the study were used to analyze mRNA expression of several cytokine targets. Cells were washed with PBS, and the pellet was frozen at -80C until RNA could be extracted. The Quick-RNA Miniprep Kit from Zymo was used to extract RNA according to manufacturer's protocol. RNase Inhibitor (New England Biolabs) was added to extracted RNA for further preservation. RNA was measured via Qubit with reagents for broad-range RNA detection (Thermo Fisher Scientific), and RNA quality was assessed via Tape

Station. RNA was then diluted to 5ng/μL and aliquoted. Six genes (aldh1a1, aldh1a2, Tnfsf13 (BAFF), TGF-β, IL-21, and IL-6) and two house-keeping genes (B2M and HPRT) were used in the multiplexed RT-qPCR assay. The SPUD assay was also included to measure any inhibition in samples or between plates[8]. Primer pairs and probes used for each cytokine are reported in Table S5. A pooled control was created to ensure consistent results between each PCR plate. Results were normalized to the B2M and HPRT reference genes for each sample. The $2^{-\Delta\Delta CT}$ Method[9] was used for analysis of relative changes in expression. Control groups included $NOD2^{\Delta DC}$+Buffer and $CD11c^{cre}$+Buffer to avoid bias due to differences in gene expression based on genotype.

Microbiome Data Processing

We used the software fastqc[10] to identify quality of fastq reads from the MiSeq, totaling 28,983,350 demultiplexed reads. The software trimmomatic (version 0.39) was used to filter and trim data. Parameters included a sliding window of four and a cutoff quality of PHRED 20, and a cutoff of 150 base-pairs or longer in order to select for high quality reads for downstream analysis. Filtered data were processed using mothur[11] (version 1.44.2) with the developers' standard operating procedure (SOP). 9,326,492 reads were used in contig assembly, and further screening lead to 7,947,996 reads with 279,391 being unique. The SILVA database (version 132) was used for alignment and classification, resulting in the discovery of 1351 OTUs. A cutoff for all samples was set to 5000 reads, and the cutoff for individual OTUs was set to ten. OTU0078 (taxonomy) was removed from analysis due to contamination being traced back to the ZymoBIOMICS Gut Microbiome Standard (D6331). Rarefaction curves were generated with the package vegan[12] in R to ensure the depth of coverage for each sample allowed for full discovery of OTUs, and shown in Supplementary Figure S4. Raw reads are

available on the National Center for Biotechnology Information's (NCBI) Sequence Read Archive (SRA) under BioProject PRJNA751895.

Alpha and Beta Diversity, Correlation, and Random Forest Analysis

Alpha diversity was analyzed using rarified richness and the Shannon diversity index. Briefly, richness was calculated from rarified data with the package vegan[12], and Shannon diversity was estimated using the phyloseq[13] package in R. A linear mixed effects model was used to predict values of richness and Shannon to account for random effects from sampling the same mice over time by using individual mice as subject-specific random effects and experimental group as the fixed variable. Predicted values were plotted to show the mean and 95% confidence intervals. Confidence intervals were used as an alternative to p-values to assess significance of between treatment levels at any time point at the 0.05 level of significance where no overlap indicates significant difference and overlap indicates no significant difference.

Beta diversity was investigated by creating Nonmetric Multidimensional Scaling (NMDS) plots. Data from the OTU level was normalized using Cumulative Sum Scaling[14]. Ordination was performed using the Bray-Curtis dissimilarity from the vegan package and had a stress of 0.129 using 3 dimensions. Plots are shown using 95% confidence ellipsoids for each timepoint or experimental group.

Random Forests[15] was used to find the most influential taxa for separating the microbiome of each experimental group, and the R package randomForest was used[16]. The optimal number of features was determined by iteration with the tuneRF function. The ntreeTry value was set to 200, and the best mtry value was found to be 25. All OTUs, sample timepoint, and processing plate number were included in classification. The features from this analysis were represented by the mean decreasing Gini[17] of the top 10 features, but full classification and Gini coefficient for all features are provided in Supplementary Table S4. A homology search

was conducted using NCBI’s BLAST[18] with the representative fastq sequence of each selected OTU identified from RF.

Supplementary References

1. Kajikawa A, Zhang L, LaVoy A, Bumgardner S, Klaenhammer TR, Dean GA. Mucosal Immunogenicity of Genetically Modified Lactobacillus acidophilus Expressing an HIV-1 Epitope within the Surface Layer Protein. PLoS ONE. 2015;10:e0141713.

2. Kajikawa A, Zhang L, Long J, Nordone S, Stoeker L, LaVoy A, et al. Construction and Immunological Evaluation of Dual Cell Surface Display of HIV-1 Gag and Salmonella enterica Serovar Typhimurium FliC in Lactobacillus acidophilus for Vaccine Delivery. Clin Vaccine Immunol. 2012;19:1374–81.

3. Goh YJ, Azcï¿½rate-Peril MA, O’Flaherty S, Durmaz E, Valence F, Jardin J, et al. Development and Application of a upp-Based Counterselective Gene Replacement System for the Study of the S-Layer Protein SlpX of Lactobacillus acidophilus NCFM. Appl Environ Microbiol. 2009;75:3093–105.

4. Stoeker L, Nordone S, Gunderson S, Zhang L, Kajikawa A, LaVoy A, et al. Assessment of Lactobacillus gasseri as a candidate oral vaccine vector. Clin Vaccine Immunol. 2011;18:1834–44.

5. Bumgardner SA, Zhang L, LaVoy AS, Andre B, Frank CB, Kajikawa A, et al. Nod2 is required for antigen-specific humoral responses against antigens orally delivered using a recombinant Lactobacillus vaccine platform. PLoS ONE. 2018;13:e0196950.

6. Kim D, Kim Y-G, Seo S-U, Kim D-J, Kamada N, Prescott D, et al. Nod2-mediated recognition of the microbiota is critical for mucosal adjuvant activity of cholera toxin. Nat Med. 2016;22:524–30.

7. Zanello G, Goethel A, Rouquier S, Prescott D, Robertson SJ, Maisonneuve C, et al. The Cytosolic Microbial Receptor Nod2 Regulates Small Intestinal Crypt Damage and Epithelial Regeneration following T Cell-Induced Enteropathy. J Immunol. 2016;197:345–55.

8. Nolan T, Hands RE, Ogunkolade W, Bustin SA. SPUD: A quantitative PCR assay for the detection of inhibitors in nucleic acid preparations. Analytical Biochemistry. 2006;351:308–10.

9. Livak KJ, Schmittgen TD. Analysis of Relative Gene Expression Data Using Real-Time Quantitative PCR and the $2^{-\Delta\Delta CT}$ Method. Methods. 2001;25:402–8.

10. FastQC. 2015. https://qubeshub.org/resources/fastqc.

11. Schloss PD, Westcott SL, Ryabin T, Hall JR, Hartmann M, Hollister EB, et al. Introducing mothur: Open-Source, Platform-Independent, Community-Supported Software for Describing and Comparing Microbial Communities. AEM. 2009;75:7537–41.

12. Oksanen J. vegan: Community Ecology Package. 2014. https://cran.r-project.org, https://github.com/vegandevs/vegan.

13. McMurdie PJ, Holmes S. phyloseq: An R Package for Reproducible Interactive Analysis and Graphics of Microbiome Census Data. PLOS ONE. 2013;8:e61217.

14. Paulson JN, Stine OC, Bravo HC, Pop M. Differential abundance analysis for microbial marker-gene surveys. Nat Methods. 2013;10:1200–2.

15. Breiman L. Random Forests. Machine Learning. 2001;45:5–32.

16. Breiman L, Cutler A. Breiman and Cutler's Random Forests for Classification and Regression: Package 'randomForest.' 2018.

17. Hastie T, Friedman JH, Tibshirani R. The Elements of Statistical Learning: Data Mining, Inference, and Prediction; Second Edition. 2009.

18. Altschul S, Madden TL, Schaffer AA, Zhang J, Zhange Z, Miller W, et al. Gapped BLAST and PSI-BLAST: a new generation of protein database search programs. Nucleic Acids Research. 1997;25:3389–402.

Supplementary Figure Legends

Supplementary Figure S1. Changes in relative abundances of bacteria over time. Relative abundance plots for each group are shown at the Family (a) and Phylum (b) level for each timepoint during the study.

Supplementary Figure S2. Changes in beta-diversity within groups over time. NMDS ordinations with data separated by experimental groups with ellipsoids representing the 95% confidence intervals for each timepoint (week) within that group. The three columns show the projections of NMDS1, NMDS2, and NMDS3, with NMDS1 and NMDS2 in column 1, NMDS1 and NMDS3 in column 2, and NMDS2 and NMDS3 in column 3.

Supplementary Figure S3. Median OOB errors of Random Forests. Out-of-bag (OOB) tuning model shows the error rates (y-axis) while iterating over 100 features (x-axis). The respective feature number with the lowest median error rate was chosen for used in the Random Forest model.

Supplementary Figure S4. Rarefaction curve of OTUs. Samples are each represented by colored lines, with OTU counts per sample on the X-axis and number of reads per sample on the y-axis. Plateauing of lines indicate a lower chance for OTU discovery based on the number of reads.

Supplementary Figure S5. Expression of $OVA_{323-339}$ by LaOVA. Histograms (a) and dot plots (b-e) show the expression of $OVA_{323-339}$ by LaOVA, but not NCK1895. LaOVA plots are shown in (b) with an anti-OVA antibody and secondary antibody conjugated with FITC, and (c) as unstained. NCK1895 plots are similarly shown in (d) with anti-OVA and FITC-secondary antibodies, and (e) as unstained.

Supplementary Figures:

Supplementary Figure S1

a Family

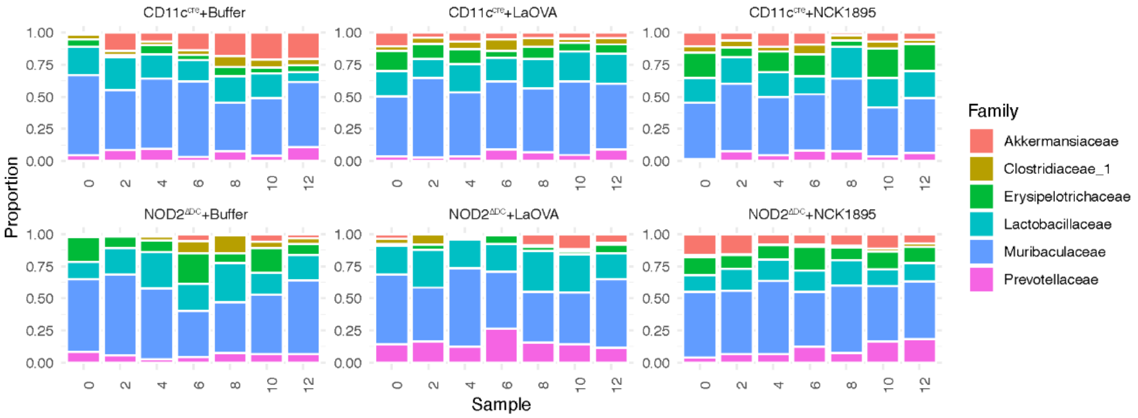

b Phylum

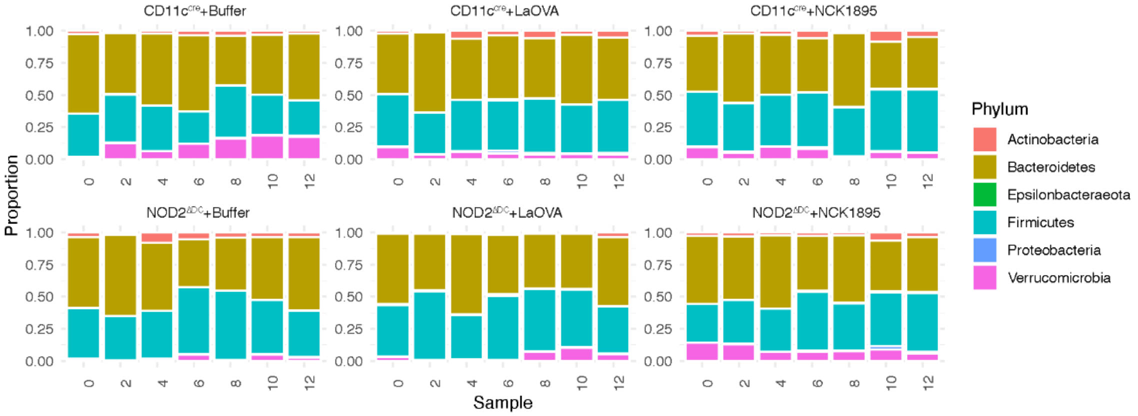

Supplementary Figure S2

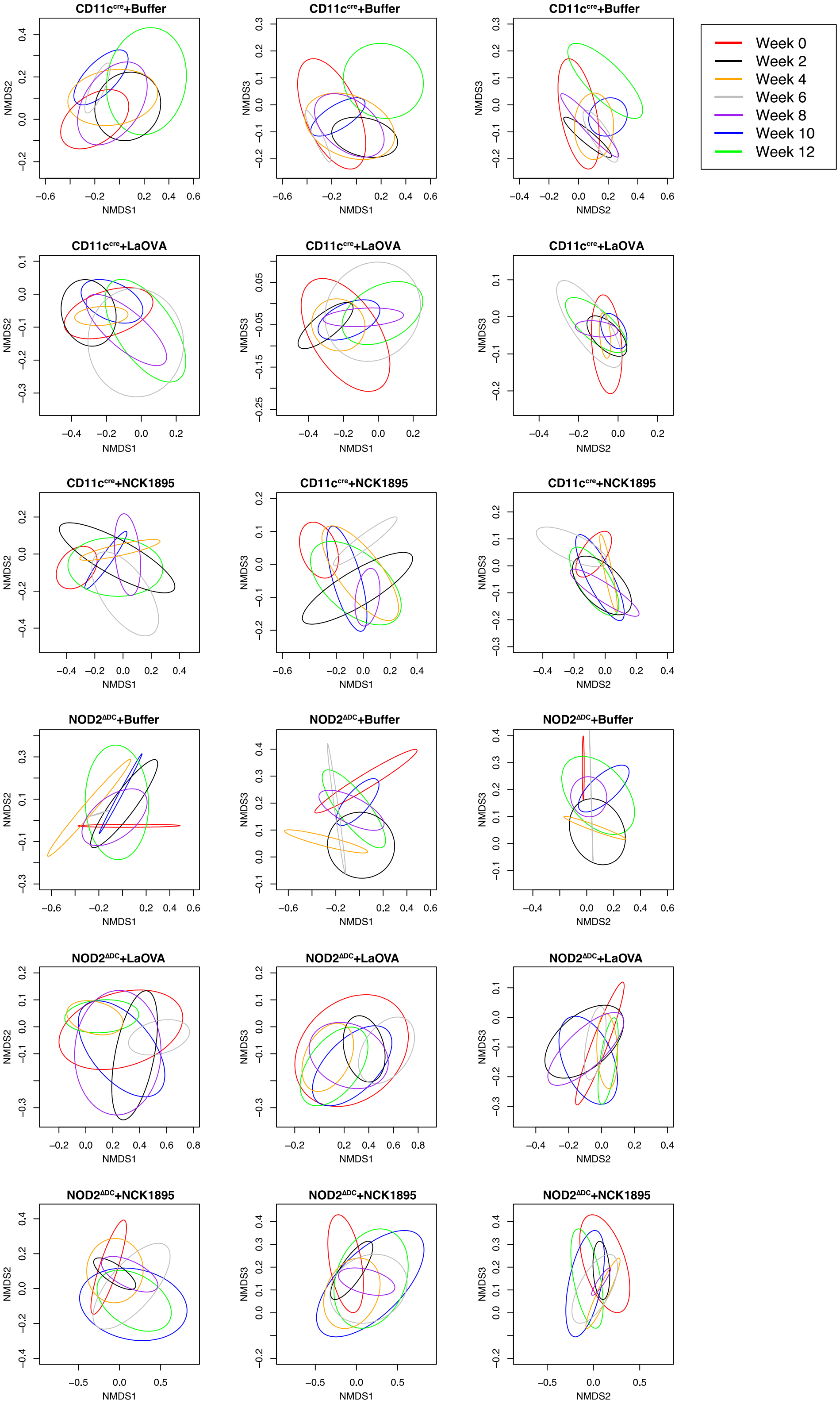

Supplementary Figure S3

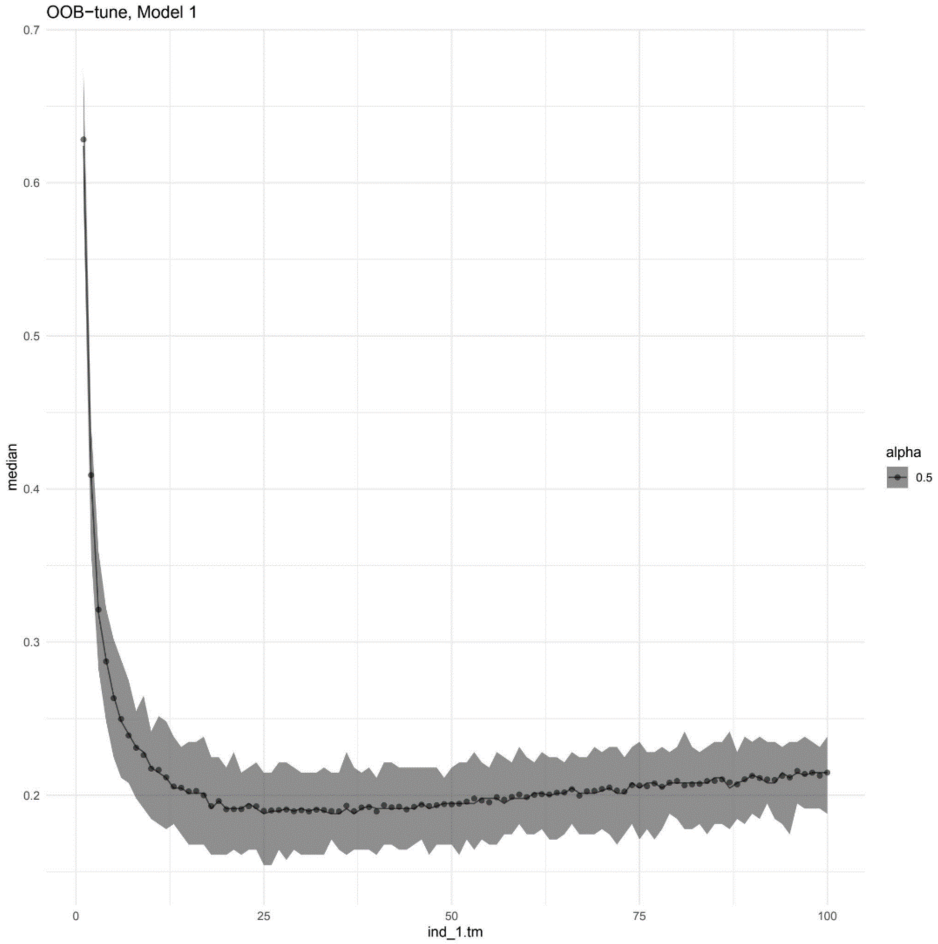

Supplementary Figure S4

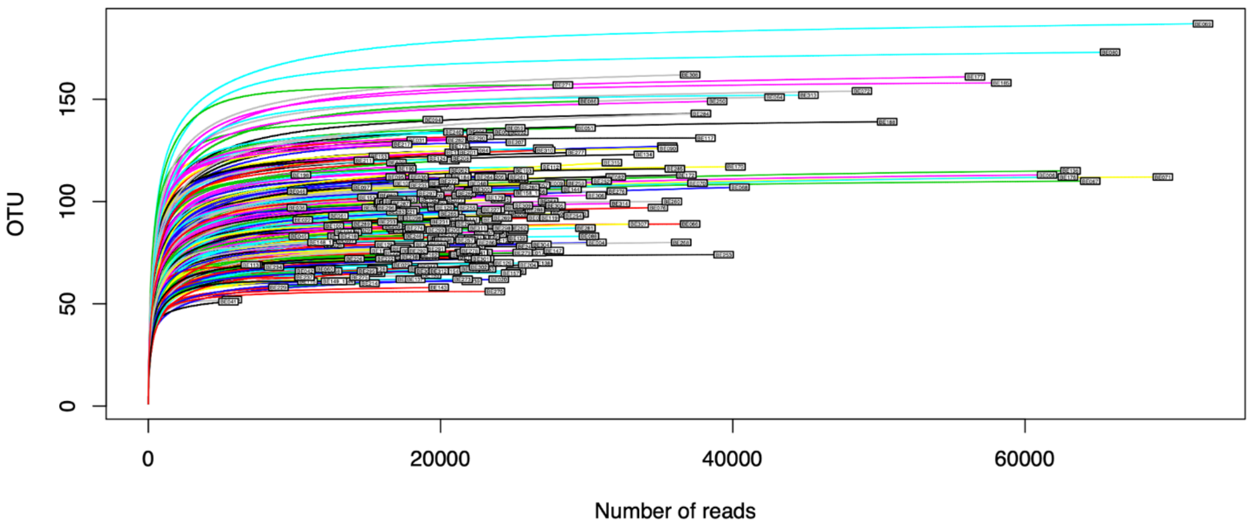

Supplementary Figure S5

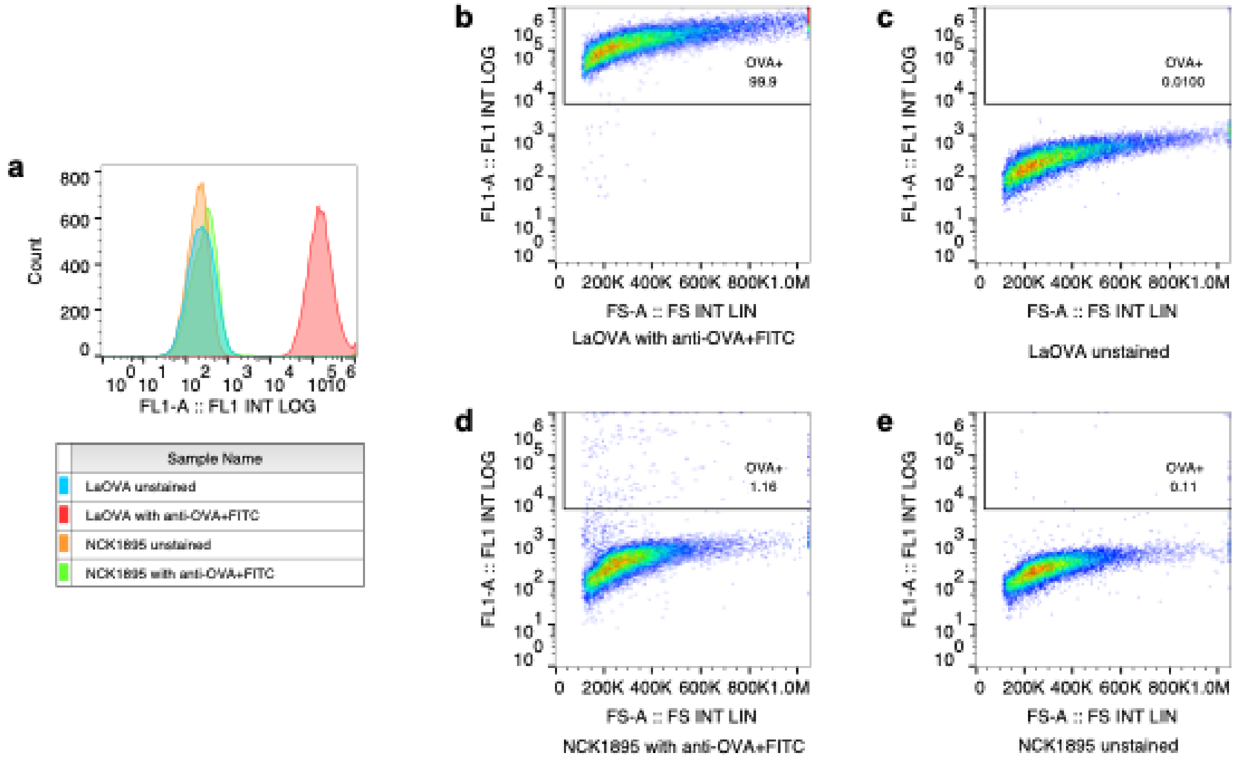

Supplementary Tables:

Supplementary Table S1. Adjusted P-values for the pairwise comparison of ELISA endpoint titers between experimental groups for each timepoint in the study. The Kruskal-Wallis test of analysis of variance was followed by Dunn's multiple comparison post-hoc test since data was not normally distributed. The Benjamini-Hochberg method was used to adjust p-values for multiple testing.

OVA-specific Fecal IgA

Comparison_1	Comparison_2	2	4	6	8	10	12
CD11c_Cre+ Buffer	CD11c_Cre+ LaOVA	0.2794	0.0106	0.0119	0.0007	0.0029	0.0025
CD11c_Cre+ Buffer	CD11c_Cre+ NCK1895	0.5000	0.5000	0.5000	0.5000	0.4442	0.5337
CD11c_Cre+ LaOVA	CD11c_Cre+ NCK1895	0.2162	0.0046	0.0065	0.0008	0.0033	0.0040
CD11c_Cre+ Buffer	NOD2_DC_KO+ Buffer	0.5185	0.5185	0.5185	0.5185	0.5000	0.5942
CD11c_Cre+ LaOVA	NOD2_DC_KO+ Buffer	0.2147	0.0072	0.0088	0.0014	0.0032	0.0069
CD11c_Cre+ NCK1895	NOD2_DC_KO+ Buffer	0.5385	0.5385	0.5385	0.5385	0.4236	0.5245
CD11c_Cre+ Buffer	NOD2_DC_KO+ LaOVA	0.4052	0.1252	0.3723	0.3520	0.4755	0.5547
CD11c_Cre+ LaOVA	NOD2_DC_KO+ LaOVA	0.3985	0.1002	0.0269	0.0028	0.0033	0.0038
CD11c_Cre+ NCK1895	NOD2_DC_KO+ LaOVA	0.3784	0.1163	0.3479	0.3492	0.5373	0.5176
NOD2_DC_KO+ Buffer	NOD2_DC_KO+ LaOVA	0.3773	0.1310	0.3507	0.3512	0.4537	0.5439
CD11c_Cre+ Buffer	NOD2_DC_KO+ NCK1895	0.5600	0.5600	0.5600	0.4449	0.5185	0.5000
CD11c_Cre+ LaOVA	NOD2_DC_KO+ NCK1895	0.2342	0.0062	0.0078	0.0021	0.0029	0.0031
CD11c_Cre+ NCK1895	NOD2_DC_KO+ NCK1895	0.5833	0.5833	0.5833	0.4332	0.4324	0.4904
NOD2_DC_KO+ Buffer	NOD2_DC_KO+ NCK1895	0.6087	0.6087	0.6087	0.4249	0.5385	0.5687
NOD2_DC_KO+ LaOVA	NOD2_DC_KO+ NCK1895	0.3983	0.1247	0.3684	0.4337	0.4626	0.5082
CD11c_Cre+ Buffer	NOD2_fl_CD11c_Cre+ LaOVA	0.3088	0.6364	0.0248	0.0278	0.0149	0.0036
CD11c_Cre+ LaOVA	NOD2_fl_CD11c_Cre+ LaOVA	0.5997	0.0092	0.3586	0.1104	0.2728	0.4947
CD11c_Cre+ NCK1895	NOD2_fl_CD11c_Cre+ LaOVA	0.2489	0.6667	0.0272	0.0328	0.0383	0.0081
NOD2_DC_KO+ Buffer	NOD2_fl_CD11c_Cre+ LaOVA	0.2224	0.7000	0.0349	0.0383	0.0246	0.0145
NOD2_DC_KO+ LaOVA	NOD2_fl_CD11c_Cre+ LaOVA	0.4020	0.1342	0.1072	0.0997	0.0372	0.0065
NOD2_DC_KO+ NCK1895	NOD2_fl_CD11c_Cre+ LaOVA	0.2738	0.7368	0.0295	0.0690	0.0184	0.0039
CD11c_Cre+ Buffer	NOD2_fl/fl+ LaOVA	0.0161	0.0124	0.0138	0.0071	0.0036	0.0049
CD11c_Cre+ LaOVA	NOD2_fl/fl+ LaOVA	0.1616	0.3232	0.6060	0.1423	0.4506	0.4928

CD11c_Cre+ NCK1895	NOD2_fl/fl+ LaOVA	0.0097	0.0129	0.0063	0.0100	0.0078	0.0040
NOD2_DC_KO+ Buffer	NOD2_fl/fl+ LaOVA	0.0145	0.0196	0.0073	0.0157	0.0053	0.0071
NOD2_DC_KO+ LaOVA	NOD2_fl/fl+ LaOVA	0.0289	0.2361	0.0227	0.0359	0.0064	0.0034
NOD2_DC_KO+ NCK1895	NOD2_fl/fl+ LaOVA	0.0145	0.0145	0.0084	0.0292	0.0034	0.0025
NOD2_fl_CD11c_Cre+ LaOVA	NOD2_fl/fl+ LaOVA	0.2003	0.0166	0.3484	0.4311	0.4777	0.5075

OVA-specific Vaginal IgA

Comparison_1	Comparison_2	2	4	6	8	10	12
CD11c_Cre+ Buffer	CD11c_Cre+ LaOVA	0.4418	0.5398	0.0010	0.0062	0.0010	0.0188
CD11c_Cre+ Buffer	CD11c_Cre+ NCK1895	0.5000	0.5000	0.5000	0.5000	0.5000	0.5000
CD11c_Cre+ LaOVA	CD11c_Cre+ NCK1895	0.3503	0.4289	0.0005	0.0071	0.0007	0.0104
CD11c_Cre+ Buffer	NOD2_DC_KO+ Buffer	0.5185	0.5185	0.5185	0.5185	0.5185	0.5185
CD11c_Cre+ LaOVA	NOD2_DC_KO+ Buffer	0.3571	0.3985	0.0011	0.0103	0.0014	0.0115
CD11c_Cre+ NCK1895	NOD2_DC_KO+ Buffer	0.5385	0.5385	0.5385	0.5385	0.5385	0.5385
CD11c_Cre+ Buffer	NOD2_DC_KO+ LaOVA	0.5600	0.5600	0.5794	0.2435	0.1420	0.1350
CD11c_Cre+ LaOVA	NOD2_DC_KO+ LaOVA	0.5050	1.0000	0.0015	0.0433	0.0221	0.1316
CD11c_Cre+ NCK1895	NOD2_DC_KO+ LaOVA	0.5833	0.5833	0.5225	0.2484	0.1401	0.1185
NOD2_DC_KO+ Buffer	NOD2_DC_KO+ LaOVA	0.6087	0.6087	0.5106	0.2597	0.1552	0.1193
CD11c_Cre+ Buffer	NOD2_DC_KO+ NCK1895	0.6364	0.3712	0.5600	0.3958	0.5600	0.5600
CD11c_Cre+ LaOVA	NOD2_DC_KO+ NCK1895	0.3853	0.3531	0.0007	0.0250	0.0011	0.0156
CD11c_Cre+ NCK1895	NOD2_DC_KO+ NCK1895	0.6667	0.3658	0.5833	0.3882	0.5833	0.5833
NOD2_DC_KO+ Buffer	NOD2_DC_KO+ NCK1895	0.7000	0.3671	0.6087	0.3850	0.6087	0.6087
NOD2_DC_KO+ LaOVA	NOD2_DC_KO+ NCK1895	0.7368	0.3998	0.5574	0.3950	0.1508	0.1259
CD11c_Cre+ Buffer	NOD2_fl_CD11c_Cre+ LaOVA	0.7778	0.4156	0.0283	0.0247	0.0134	0.0129
CD11c_Cre+ LaOVA	NOD2_fl_CD11c_Cre+ LaOVA	0.4281	0.4050	0.1978	0.4156	0.2552	0.5847
CD11c_Cre+ NCK1895	NOD2_fl_CD11c_Cre+ LaOVA	0.8235	0.4046	0.0293	0.0290	0.0144	0.0109
NOD2_DC_KO+ Buffer	NOD2_fl_CD11c_Cre+ LaOVA	0.8750	0.4041	0.0375	0.0372	0.0197	0.0154
NOD2_DC_KO+ LaOVA	NOD2_fl_CD11c_Cre+ LaOVA	0.9333	0.4618	0.0633	0.1919	0.1738	0.1162
NOD2_DC_KO+ NCK1895	NOD2_fl_CD11c_Cre+ LaOVA	1.0000	0.5910	0.0330	0.0905	0.0164	0.0127

CD11c_Cre+ Buffer	NOD2_fl/fl+ LaOVA	0.4655	0.4903	0.5108	0.5600	0.3355	0.1393
CD11c_Cre+ LaOVA	NOD2_fl/fl+ LaOVA	0.5823	0.3864	0.0010	0.0053	0.0014	0.1051
CD11c_Cre+ NCK1895	NOD2_fl/fl+ LaOVA	0.2300	0.4703	0.4716	0.5833	0.3196	0.1208
NOD2_DC_KO+ Buffer	NOD2_fl/fl+ LaOVA	0.2464	0.4709	0.4634	0.6087	0.3247	0.1164
NOD2_DC_KO+ LaOVA	NOD2_fl/fl+ LaOVA	0.9310	0.5884	0.5614	0.2288	0.2831	0.6171
NOD2_DC_KO+ NCK1895	NOD2_fl/fl+ LaOVA	0.2875	0.5686	0.4964	0.3983	0.3356	0.1294
NOD2_fl_CD11c_Cre+ LaOVA	NOD2_fl/fl+ LaOVA	0.3833	0.5962	0.0368	0.0220	0.0332	0.1365

OVA-specific Serum IgG

Comparison_1	Comparison_2	2	4	6	8	10	12
CD11c_Cre+ Buffer	CD11c_Cre+ LaOVA	0.5187	0.4475	0.2473	0.0293	0.0117	0.0137
CD11c_Cre+ Buffer	CD11c_Cre+ NCK1895	0.5000	0.5000	0.3980	0.2130	0.3856	0.3064
CD11c_Cre+ LaOVA	CD11c_Cre+ NCK1895	0.4158	0.1906	0.1662	0.0040	0.0097	0.0076
CD11c_Cre+ Buffer	NOD2_DC_KO+ Buffer	0.5185	0.5185	0.2425	0.1449	0.4209	0.2012
CD11c_Cre+ LaOVA	NOD2_DC_KO+ Buffer	0.4167	0.1927	0.1431	0.0068	0.0134	0.0085
CD11c_Cre+ NCK1895	NOD2_DC_KO+ Buffer	0.5385	0.5385	0.3650	0.3780	0.4695	0.4070
CD11c_Cre+ Buffer	NOD2_DC_KO+ LaOVA	0.5600	0.4409	0.3896	0.3291	0.4527	0.4067
CD11c_Cre+ LaOVA	NOD2_DC_KO+ LaOVA	0.5835	0.2907	0.1479	0.0096	0.0155	0.0070
CD11c_Cre+ NCK1895	NOD2_DC_KO+ LaOVA	0.5833	0.4028	0.4954	0.3549	0.3739	0.4399
NOD2_DC_KO+ Buffer	NOD2_DC_KO+ LaOVA	0.6087	0.3793	0.3648	0.2937	0.3931	0.3219
CD11c_Cre+ Buffer	NOD2_DC_KO+ NCK1895	0.6364	0.5600	0.2474	0.2256	0.4465	0.4271
CD11c_Cre+ LaOVA	NOD2_DC_KO+ NCK1895	0.4536	0.2860	0.2970	0.0060	0.0105	0.0078
CD11c_Cre+ NCK1895	NOD2_DC_KO+ NCK1895	0.6667	0.5833	0.3687	0.5000	0.4535	0.3987
NOD2_DC_KO+ Buffer	NOD2_DC_KO+ NCK1895	0.7000	0.6087	0.5000	0.3925	0.4406	0.3412
NOD2_DC_KO+ LaOVA	NOD2_DC_KO+ NCK1895	0.7368	0.4315	0.3837	0.3697	0.4342	0.4768
CD11c_Cre+ Buffer	NOD2_fl_CD11c_Cre+ LaOVA	0.7778	0.3939	0.2400	0.1302	0.0372	0.0265
CD11c_Cre+ LaOVA	NOD2_fl_CD11c_Cre+ LaOVA	0.4990	0.2759	0.5145	0.3120	0.4007	0.4424
CD11c_Cre+ NCK1895	NOD2_fl_CD11c_Cre+ LaOVA	0.8235	0.3461	0.1335	0.0251	0.0173	0.0073
NOD2_DC_KO+ Buffer	NOD2_fl_CD11c_Cre+ LaOVA	0.8750	0.3468	0.1342	0.0194	0.0288	0.0060

NOD2_DC_KO+ LaOVA	NOD2_fl_CD11c_Cre+ LaOVA	0.9333	0.6204	0.1264	0.0386	0.0462	0.0130
NOD2_DC_KO+ NCK1895	NOD2_fl_CD11c_Cre+ LaOVA	1.0000	0.3653	0.1963	0.0293	0.0300	0.0127
CD11c_Cre+ Buffer	NOD2_fl/fl+ LaOVA	0.0297	0.1793	0.3946	0.2160	0.0369	0.0271
CD11c_Cre+ LaOVA	NOD2_fl/fl+ LaOVA	0.1358	0.4395	0.3774	0.1349	0.2697	0.4130
CD11c_Cre+ NCK1895	NOD2_fl/fl+ LaOVA	0.0189	0.1587	0.2161	0.0394	0.0194	0.0066
NOD2_DC_KO+ Buffer	NOD2_fl/fl+ LaOVA	0.0272	0.1792	0.1336	0.0296	0.0274	0.0061
NOD2_DC_KO+ LaOVA	NOD2_fl/fl+ LaOVA	0.0594	0.4225	0.2116	0.0875	0.0482	0.0119
NOD2_DC_KO+ NCK1895	NOD2_fl/fl+ LaOVA	0.0236	0.1852	0.1594	0.0433	0.0289	0.0123
NOD2_fl_CD11c_Cre+ LaOVA	NOD2_fl/fl+ LaOVA	0.0315	0.3977	0.3615	0.3292	0.4574	0.4385

Supplementary Table S2. Adjusted P-values for the pairwise comparison of ELISpot spot forming units between experimental groups for each tissue type that was sampled. The Kruskal-Wallis test of analysis of variance was followed by Dunn’s multiple comparison post-hoc test since data was also not normally distributed. The Benjamini-Hochberg method was used to adjust p-values for multiple testing.

OVA-specific SFU

**comparisons_1**	**comparisons_2**	**FRT**	**LI**	**MLN**	**PP**	**Sp**
CD11c_Cre+Buffer	CD11c_Cre+LaOVA	0.0201	0.1132	0.0025	0.1689	0.0961
CD11c_Cre+Buffer	CD11c_Cre+NCK1895	0.5000	0.5000	0.5000	0.5000	0.5000
CD11c_Cre+LaOVA	CD11c_Cre+NCK1895	0.0301	0.1258	0.0023	0.1653	0.0872
CD11c_Cre+Buffer	NOD2_DC_KO+Buffer	0.5185	0.5185	0.5185	0.5185	0.5185
CD11c_Cre+LaOVA	NOD2_DC_KO+Buffer	0.0373	0.1508	0.0033	0.1692	0.0908
CD11c_Cre+NCK1895	NOD2_DC_KO+Buffer	0.5385	0.5385	0.5385	0.5385	0.5385
CD11c_Cre+Buffer	NOD2_DC_KO+LaOVA	0.4117	0.5600	0.5600	0.5600	0.3002
CD11c_Cre+LaOVA	NOD2_DC_KO+LaOVA	0.0361	0.1415	0.0050	0.1830	0.2606
CD11c_Cre+NCK1895	NOD2_DC_KO+LaOVA	0.4346	0.5833	0.5833	0.5833	0.2681
NOD2_DC_KO+Buffer	NOD2_DC_KO+LaOVA	0.4006	0.6087	0.6087	0.6087	0.2766
CD11c_Cre+Buffer	NOD2_DC_KO+NCK1895	0.5600	0.6364	0.6364	0.6364	0.5600
CD11c_Cre+LaOVA	NOD2_DC_KO+NCK1895	0.0603	0.1618	0.0031	0.1771	0.1018
CD11c_Cre+NCK1895	NOD2_DC_KO+NCK1895	0.5833	0.6667	0.6667	0.6667	0.5833
NOD2_DC_KO+Buffer	NOD2_DC_KO+NCK1895	0.6087	0.7000	0.7000	0.7000	0.6087
NOD2_DC_KO+LaOVA	NOD2_DC_KO+NCK1895	0.4601	0.7368	0.7368	0.7368	0.2838
CD11c_Cre+Buffer	NOD2_fl_CD11c_Cre+LaOVA	0.0371	0.0095	0.0265	0.0034	0.1005
CD11c_Cre+LaOVA	NOD2_fl_CD11c_Cre+LaOVA	0.4075	0.2267	0.3550	0.0644	0.5768
CD11c_Cre+NCK1895	NOD2_fl_CD11c_Cre+LaOVA	0.0424	0.0127	0.0280	0.0029	0.0803
NOD2_DC_KO+Buffer	NOD2_fl_CD11c_Cre+LaOVA	0.0550	0.0205	0.0360	0.0045	0.0929

NOD2_DC_KO+LaOVA	NOD2_fl_CD11c_Cre+LaOVA	0.0997	0.0190	0.0302	0.0067	0.2970
NOD2_DC_KO+NCK1895	NOD2_fl_CD11c_Cre+LaOVA	0.0494	0.0381	0.0311	0.0039	0.0876
CD11c_Cre+Buffer	NOD2_fl/fl+LaOVA	0.4578	0.4638	0.5484	0.1976	0.0456
CD11c_Cre+LaOVA	NOD2_fl/fl+LaOVA	0.0508	0.2307	0.0030	0.7700	0.3310
CD11c_Cre+NCK1895	NOD2_fl/fl+LaOVA	0.4905	0.4928	0.5002	0.1887	0.0248
NOD2_DC_KO+Buffer	NOD2_fl/fl+LaOVA	0.4753	0.4746	0.4919	0.1561	0.0331
NOD2_DC_KO+LaOVA	NOD2_fl/fl+LaOVA	0.6037	0.5257	0.5876	0.2223	0.0836
NOD2_DC_KO+NCK1895	NOD2_fl/fl+LaOVA	0.5283	0.5632	0.5315	0.2076	0.0372
NOD2_fl_CD11c_Cre+LaOVA	NOD2_fl/fl+LaOVA	0.0968	0.0201	0.0452	0.0552	0.3093

Total_IgA SFU

**comparisons_1**	**comparisons_2**	**FRT**	**LI**	**MLN**	**PP**	**Sp**
CD11c_Cre+Buffer	CD11c_Cre+LaOVA	0.3502	0.3926	0.2792	0.0567	0.4521
CD11c_Cre+Buffer	CD11c_Cre+NCK1895	0.3839	0.2103	0.2009	0.4335	0.3598
CD11c_Cre+LaOVA	CD11c_Cre+NCK1895	0.2172	0.1389	0.3701	0.0612	0.3740
CD11c_Cre+Buffer	NOD2_DC_KO+Buffer	0.0599	0.0533	0.3935	0.0522	0.3665
CD11c_Cre+LaOVA	NOD2_DC_KO+Buffer	0.1544	0.0689	0.2482	0.5118	0.3526
CD11c_Cre+NCK1895	NOD2_DC_KO+Buffer	0.0688	0.1932	0.1534	0.0595	0.1838
CD11c_Cre+Buffer	NOD2_DC_KO+LaOVA	0.4312	0.1006	0.2823	0.0637	0.3322
CD11c_Cre+LaOVA	NOD2_DC_KO+LaOVA	0.2932	0.0659	0.1207	0.3335	0.3174
CD11c_Cre+NCK1895	NOD2_DC_KO+LaOVA	0.4083	0.3198	0.0565	0.1335	0.2231
NOD2_DC_KO+Buffer	NOD2_DC_KO+LaOVA	0.0646	0.2924	0.3584	0.3414	0.4658
CD11c_Cre+Buffer	NOD2_DC_KO+NCK1895	0.0653	0.1515	0.3622	0.4379	0.4967
CD11c_Cre+LaOVA	NOD2_DC_KO+NCK1895	0.1555	0.1135	0.3789	0.0621	0.4434
CD11c_Cre+NCK1895	NOD2_DC_KO+NCK1895	0.1108	0.4083	0.2753	0.4887	0.2910
NOD2_DC_KO+Buffer	NOD2_DC_KO+NCK1895	0.4337	0.2223	0.3058	0.0617	0.3549
NOD2_DC_KO+LaOVA	NOD2_DC_KO+NCK1895	0.0732	0.4007	0.1916	0.1340	0.3275
CD11c_Cre+Buffer	NOD2_fl_CD11c_Cre+LaOVA	0.2323	0.0272	0.0933	0.0666	0.4598
CD11c_Cre+LaOVA	NOD2_fl_CD11c_Cre+LaOVA	0.4002	0.0171	0.0208	0.3137	0.3683
CD11c_Cre+NCK1895	NOD2_fl_CD11c_Cre+LaOVA	0.1666	0.1317	0.0161	0.1419	0.2844
NOD2_DC_KO+Buffer	NOD2_fl_CD11c_Cre+LaOVA	0.2193	0.4384	0.1839	0.3170	0.4462
NOD2_DC_KO+LaOVA	NOD2_fl_CD11c_Cre+LaOVA	0.2253	0.2323	0.3011	0.4983	0.4419
NOD2_DC_KO+NCK1895	NOD2_fl_CD11c_Cre+LaOVA	0.2245	0.1842	0.0564	0.1422	0.4230
CD11c_Cre+Buffer	NOD2_fl/fl+LaOVA	0.0751	0.2803	0.2810	0.4956	0.4122
CD11c_Cre+LaOVA	NOD2_fl/fl+LaOVA	0.2099	0.2088	0.4416	0.0549	0.3416
CD11c_Cre+NCK1895	NOD2_fl/fl+LaOVA	0.0531	0.3499	0.3125	0.4470	0.1215
NOD2_DC_KO+Buffer	NOD2_fl/fl+LaOVA	0.3800	0.1057	0.2565	0.0567	0.4588
NOD2_DC_KO+LaOVA	NOD2_fl/fl+LaOVA	0.0718	0.2002	0.1066	0.0646	0.4356
NOD2_DC_KO+NCK1895	NOD2_fl/fl+LaOVA	0.4079	0.2596	0.3947	0.4492	0.3949
NOD2_fl_CD11c_Cre+LaOVA	NOD2_fl/fl+LaOVA	0.2856	0.0578	0.0277	0.0688	0.3451

Supplementary Table S3. Adjusted P-values for the pairwise comparison of RT-qPCR results between experimental groups for each cytokine. The Kruskal-Wallis test of analysis of variance was followed by Dunn's multiple comparison post-hoc test, and the Benjamini-Hochberg method was used to account for multiple testing.

Mesenteric Lymph Nodes

comparisons_1	comparisons_2	aladh1a1	aladh1a2	BAFF	IL21	IL6	TGFB
KO_LaOVA	KO_NCK1895	0.3801	0.5130	0.3898	0.1946	0.4594	0.4678
KO_LaOVA	WT_LaOVA	0.0087	0.4738	0.0756	0.1972	0.8019	0.0057
KO_NCK1895	WT_LaOVA	0.0100	0.4393	0.0958	0.0552	0.4739	0.0071
KO_LaOVA	WT_NCK1895	0.0215	1.0000	0.4678	0.3451	0.4444	0.0048
KO_NCK1895	WT_NCK1895	0.0170	0.5921	0.4441	0.2159	0.3806	0.0089
WT_LaOVA	WT_NCK1895	0.3956	0.7144	0.1128	0.2436	0.4537	0.3898

Peyer's Patches

comparisons_1	comparisons_2	aladh1a1	aladh1a2	BAFF	IL21	IL6	TGFB
KO_LaOVA	KO_NCK1895	0.2304	0.5155	0.1800	0.2323	0.4394	0.0953
KO_LaOVA	WT_LaOVA	0.0550	0.3262	0.0076	0.2629	0.1998	0.2817
KO_NCK1895	WT_LaOVA	0.1572	0.5384	0.0595	0.4899	0.2186	0.0378
KO_LaOVA	WT_NCK1895	0.2240	0.3750	0.1116	0.1419	0.0381	0.0034
KO_NCK1895	WT_NCK1895	0.1192	0.9242	0.0321	0.2840	0.0376	0.0864
WT_LaOVA	WT_NCK1895	0.0118	0.4594	0.0002	0.4112	0.1520	0.0009

Additional File 3: Supplementary Table S4. [Excel file titled Table_S4]. Gini coefficients of importance for features from the RF model. Table represents and expansion of the Gini coefficients shown in Figure 6, with OTU listed in the first column followed by taxonomic classification. The last column shows the mean Gini coefficient of importance for that feature.

Table S5. Primer and Probe pair sequences for cytokines used in RT-qPCR.

Gene	Primer Sequences	Probe Sequence
HPRT<br>Exon: 6-7	5'-AAC AAA GTC TGG CCT GTA TCC-3'<br>5'-CCC CAA AAT GGT TAA GGT TGC-3'	5'-/56-FAM/CTT GCT GGT/ZEN/GAA AAG GAC CTC TCG GAA/3IABkFQ/-3'
B2m<br>Exon: 1-2	5'-GGG TGG AAC TGT GTT ACG TAG-3'<br>5'-TGG TCT TTC TGG TGC TTG TC-3'	5'-/56-FAM/CCG GAG AAT/ZEN/GGG AAG CCG AAC ATA C/3IABkFQ/-3'
Aldh1a1<br>Exon: 11-13	5'-ACC CAG TTC TCT TCC ATT TCC-3'<br>5'-CAT CAC TGT GTC ATC TGC TCT-3'	5'-/56-FAM/ACA CTG CCC/ZEN/AAC AAT TCC TGC TAC T/3IABkFQ/-3'
Aldh1a2<br>Exon: 8-9	5'-CAC TGG CCT TGG TTG AAG A-3'<br>5'-GAA GTA ACC TGA AGA GAG TGA CC-3'	5'-/5HEX/AGA TGC TGA/ZEN/CTT GGA CTA CGC TGT G/3IABkFQ/-3'
Tnfsf13b<br>(BAFF)<br>Exon: 6-7	5'-TCA TCT CCT TCT TCC AGC CT-3'<br>5'-GAC CCT GTT CCG ATG TAT TCA G-3'	5'-/56-FAM/ACA CTG CCC/ZEN/AAC AAT TCC TGC TAC T/3IABkFQ/-3'
IL21<br>Exon: 1-3	5'-GGT TTG ATG GCT TGA GTT TGG-3'<br>5'-TGA CTT GGA TCC TGA ACT TCT ATC-3'	5'-/5HEX/TGC TCA CAG/ZEN/TGC CCC TTT ACA TCT T/3IABkFQ/-3'
IL6<br>Exon: 4-5	5'-TCC TTA GCC ACT CCT TCT GT-3'<br>5'-AGC CAG AGT CCT TCA GAG A-3'	5'-/56-FAM/AGT TAA CCC/ZEN/ACA CCA CCC CAG C/3IABkFQ/-3'
TGFb1<br>Exon: 1-2	5'-CCG AAT GTC TGA CGT ATT GAA GA-3'<br>5'-GCG GAC TAC TAT GCT AAA GAG G-3'	5'-/5HEX/ATA GAT GGCZEN/GTT GTT GCG GTC CA/3IABkFQ/-3'
